# Reasoning and interpretation cognitive biases related to psychotic characteristics: An umbrella-review

**DOI:** 10.1371/journal.pone.0314965

**Published:** 2024-12-27

**Authors:** Crystal Samson, Audrey Livet, Andy Gilker, Stephane Potvin, Veronik Sicard, Tania Lecomte

**Affiliations:** 1 Département de Psychologie, Université de Montréal, Montréal, Québec, Canada; 2 Centre de recherche de l’Institut Universitaire en Santé Mentale de Montréal (CR-IUSMM), Montréal, Québec, Canada; 3 Centre de recherche du Centre Hospitalier de l’Université de Montréal (CHUM), Montréal, Québec, Canada; 4 Clinique des Jeunes Adultes Psychotiques (JAP), Montréal, Québec, Canada; 5 Département de Génie biotechnologique, Université de Sherbrooke, Sherbrooke, Québec, Canada; 6 Children’s Hospital of Eastern Ontario Research Institute, Ottawa, Ontario, Canada; Karamanoglu Mehmetbey University: Karamanoglu Mehmetbey Universitesi, TÜRKIYE

## Abstract

Cognitive biases have been studied in relation to schizophrenia and psychosis for over 50 years. Yet, the quality of the evidence linking cognitive biases and psychosis is not entirely clear. This umbrella-review examines the quality of the evidence and summarizes the effect sizes of the reasoning and interpretation cognitive biases studied in relation to psychotic characteristics (psychotic disorders, psychotic symptoms, psychotic-like experiences or psychosis risk). It also examines the evidence and the effects of psychological interventions for psychosis on cognitive biases. A systematic review of the literature was performed using the PRISMA guidelines and the GRADE system for 128 analyses extracted from 16 meta-analyses. Moderate to high-quality evidence with medium to large effect sizes were found for the following interpretation biases: externalization of cognitive events and self-serving bias, when people with psychotic symptoms were compared to control conditions. Regarding reasoning biases, moderate to high quality evidence with medium to large effect sizes were found for belief inflexibility when linked to delusion conviction and global severity in people with active delusions, although measures from the MADS, overlapping with symptoms, may have inflated effect sizes. Moderate quality evidence with medium to large effect sizes were found for jumping to conclusion biases when clinical samples with psychosis were compared to controls, when using data-gathering tasks. Other cognitive biases are not supported by quality evidence (e.g., personalizing bias, belief about disconfirmatory evidence), and certain measures (i.e., IPSAQ and ASQ) systematically found no effect or small effects. Psychological interventions (e.g., MCT) showed small effect sizes on cognitive biases, with moderate-high-quality evidence. This umbrella review brings a critical regard on the reasoning and interpretation biases and psychotic symptoms literature—although most biases linked to psychotic symptoms are supported by meta-analyses in some way, some have only demonstrated support with a specific population group (e.g., aberrant salience and hostility attribution in healthy individuals with psychotic-like experiences), whereas other biases are currently insufficiently supported by quality evidence. Future quality studies, particularly with clinical populations with psychotic symptoms, are still warranted to ascertain the psychosis-cognitive bias link for specific biases.

## Introduction

According to several theoretical models, cognitive biases, particularly reasoning and interpretation biases, are essential in psychotic symptom development and maintenance. A recent systematic review by Gaweda, encompassing 40 years of research on cognitive biases, describes several theories linking specific cognitive biases to psychotic symptoms or phenomena [1]. For instance, Freeman links reasoning biases to delusions in general, and persecutory delusions in particular [2], whereas Bentall has included attributional styles in his model of persecutory delusion formation [3]. These models, as well as the wealth of studies, including systematic reviews, on cognitive biases as linked to delusions, lead us to take for granted that the quality of the evidence is solid and that the effect size is substantial. Yet, a recent meta-analysis explored the link between childhood trauma and cognitive biases in those with psychosis [4], and concluded that the quality evidence of the studies was insufficient to currently determine which biases emerge following trauma, and whether they act as mediators in the relationship between childhood trauma and psychosis. To date, and to our knowledge, there is no umbrella review (i.e., a systematic review of meta-analyses) reporting the quality of the evidence and summarizing the effect sizes regarding interpretation and reasoning cognitive biases studies in psychosis, either linking biases to psychotic characteristics or looking at the effects of psychological intervention on cognitive biases. While systematic reviews or meta-analyses summarize the information found in individual studies, an umbrella review looks at the quality of the evidence across multiple meta-analyses and can help answer the question “How sure are we about this link or result?”. As such, the objective of this umbrella review is to examine the quality of the evidence, as well as the size of the association between interpretation and/or reasoning cognitive biases and psychotic characteristics (psychotic disorders, psychotic symptoms, psychotic-like experiences, or psychosis risk), and the effect of psychological interventions on changes in cognitive biases.

Why only focus on interpretation and reasoning biases? Beck [5] defined cognitive biases as systematic errors in thinking underlying psychopathology. Beck and Clark [6] proposed a three-stage model of information processing: 1) perception and attention, 2) interpretation and 3) recall. Rector and Beck [7] later conceptualized a cognitive explanation for delusions, auditory hallucinations, negative symptoms and thought disorders using the framework of cognitive biases. According to them, cognitive biases act in concert with the content of the individual’s belief system, increasing the individual’s psychological vulnerability to delusions. This model greatly influenced the development of cognitive behaviour therapy for psychosis [8], as well as metacognitive training (MCT [9]).

Many authors, such as Mathews and MacLeod [10], mention three categories of cognitive biases: attentional biases, interpretation biases and memory biases (or recall biases), reflecting Beck and Clark’s [6] three-stage model of information processing. It is important to consider that today, most authors distinguish reasoning biases from interpretation biases, reflecting Blanchette and Richards’ [11] differentiation of these complex cognitive processes (interpretation, reasoning, judgment and decision-making). Over the years, several published studies on cognitive biases in psychosis partly support this conceptualization, enabling several systematic reviews and meta-analyses. Nevertheless, these reviews do not all cover the same cognitive biases or report the same studies–making it difficult to ascertain if the cognitive models linking biases to psychosis hold true across multiple biases. Although a recent systematic review has attempted to include many cognitive biases measured via performance tasks [1], and therefore offer a more comprehensive review of the topic with possible links between several cognitive biases and symptoms, it was not a meta-analysis and therefore did not offer aggregated effect sizes, nor did it assess the quality of the studies. Still, all of these reviews have in common to have mostly documented reasoning and interpretation biases.

Therefore, in order to largely cover the recent literature while keeping a focus on the biases that have mostly been studied in relation to psychotic symptom development, maintenance and treatment, we chose to only focus on interpretation and reasoning biases in this umbrella review.

## Materials and methods

### Literature search strategy

This systematic review adhered to the 2020 Preferred Reporting Items for Systematic Reviews and Meta-Analyses guidelines [12] and was initiated on October 26, 2021, with no time span specified regarding the initial date of publication. An updated search was conducted on June 25th 2024, and revealed three more meta-analyses, two that did not fit inclusion criteria and one that was added. The four following databases were systematically searched: PsyNet (PsycINFO and PsycARTICLES), Medline and Web of Science. The following keywords were used: (schizophren* or psychotic or psychosis or schizotyp* or “psychotic like” or “psychosis like” or “psychosis risk” or “psychotic risk” or paranoia or hallucination* or delusion*) and (bias* or distortion or “dysfunctional attitude” or “dysfunctional thought*” or “dysfunctional thinking” or distortion* or “distorted attitude*” or “distorted thinking” or “distorted thought*”) and (“meta-analysis”).

### Inclusion and exclusion criteria

The decision to include or exclude reviews were made by consensus between authors.

### Inclusion criteria

- We included meta-analyses that investigate the association between reasoning or interpretative cognitive biases and psychotic features. Specifically, studies were selected if they:
  1. Compare cognitive bias scores between individuals with psychotic disorders, psychotic symptoms, psychotic-like experiences, or psychosis risk and a control group.
  2. Examine correlations between reasoning or interpretative cognitive bias and symptom levels.
  3. Assess the impact of psychological interventions on cognitive biases.
- To further define the scope, included meta-analyses met these additional criteria:
  1. Metanalyses containing systematic reviews.
  2. Full-text peer-reviewed publications in either English or French, with books and conference abstracts excluded.
  3. Studies involving adult populations, defined as those with a mean participant age over 18 years.
- Analogue studies (i.e., experimental designs in which the procedures or participants used are similar but not identical to the situation of interest) in which participants did not have psychotic symptoms but could have other psychotic-like experiences, were at risk for psychosis or were rated on a psychotic-like experiences scale were included.

### Exclusion criteria

- Meta-analyses about emotion recognition were excluded as these are largely considered a social cognitive deficit rather than a cognitive bias [13–16].
- Aggression bias was excluded because it refers more to a score of behavioral intention of aggressiveness than to a cognitive bias.
- Need for closure was also excluded, since it refers to the motivation to achieve finality and absoluteness in decisions, judgments, and choices, often prematurely and is typically considered an underlying motivation that can explain the jumping to conclusion bias [17], or a personality trait [17, 18].

### Data extraction

Information on nine factors were retrieved when available, namely: 1) study design, 2) outcomes (e.g., type of cognitive biases, psychotic characteristics studied), 3) effect size, 4) confidence interval, 5) consistency (homogeneity, I squared, Cochran’s Q statistic), 6) number of studies and number of participants, 8) publication bias and, 9) consideration of confounding factors (e.g., age, sex, IQ).

### Conclusions about effect sizes

The magnitude of the relation between a cognitive bias and a psychotic feature or symptom, as well as the effects of psychological interventions on cognitive biases, were determined based on the effect size estimated by the investigators [19]. In the current umbrella-review, effect size estimates were calculated using Cohen’s d, Hedge’s g, Pearson’s correlation coefficients or odds ratio. By convention [20], d/g estimates of 0.8, 0.55 and 0.2 were considered as large, moderate and small. Specifications about conclusions on effect sizes, R-values estimates included, are presented in supplementary material (S1 Table).

### Quality assessment using the GRADE system

The GRADE system was used to assess the quality of the evidence following the data extraction [21]. Exact criteria used for the Quality assessment using the GRADE system are presented in supplementary material (S2 Table). According to this assessment system, the quality of the evidence of the meta-analyses’ results can be judged based on various factors, namely: 1) the size of the sample (the larger the better, ideally over 1000); 2) the precision of effects (i.e., the confidence interval is not too wide—as in Matheson and colleagues [22], 25% higher or lower than the effect size was considered ideal); 3) homogeneity of effects across studies (i.e., consistency of results from one study to the next), and 4) publication bias (if analyzed and if present). We checked for follow-up data (if any and the length of time) for meta-analyses regarding the effects of psychological treatments on cognitive biases, but not for the studies on the relation between biases and psychotic characteristics.

We also added a specific section for confounding factors considered, which could include controlling for study biases, trial quality, as well as other variables that could influence the results. Following the recommended guidelines, no points were given for the effect size. As such, a point is given for each element of the GRADE system measured (e.g., sample size, precision, consistency, publication bias and confounding factors; and follow-up data in the case of trials), with meta-analyses being rated from poor, poor to moderate, moderate, moderate to high, high–quality evidence, to very high-quality evidence, for a maximum of 6 points. If information was missing in the article, the authors of the meta-analysis were contacted and if they did not respond within two months, we considered the information as missing and no point was given for this factor. For each meta-analysis, four of the authors rated the different components with the GRADE system. Differences were discussed and a consensus was made for a final decision. Given the stringent criteria involved, consensus was easily reached (over 95% initial agreement). The authors of this article already used the GRADE system in previous umbrella and meta-reviews [23, 24].

## Results

### Included and excluded studies

**Fig 1:**
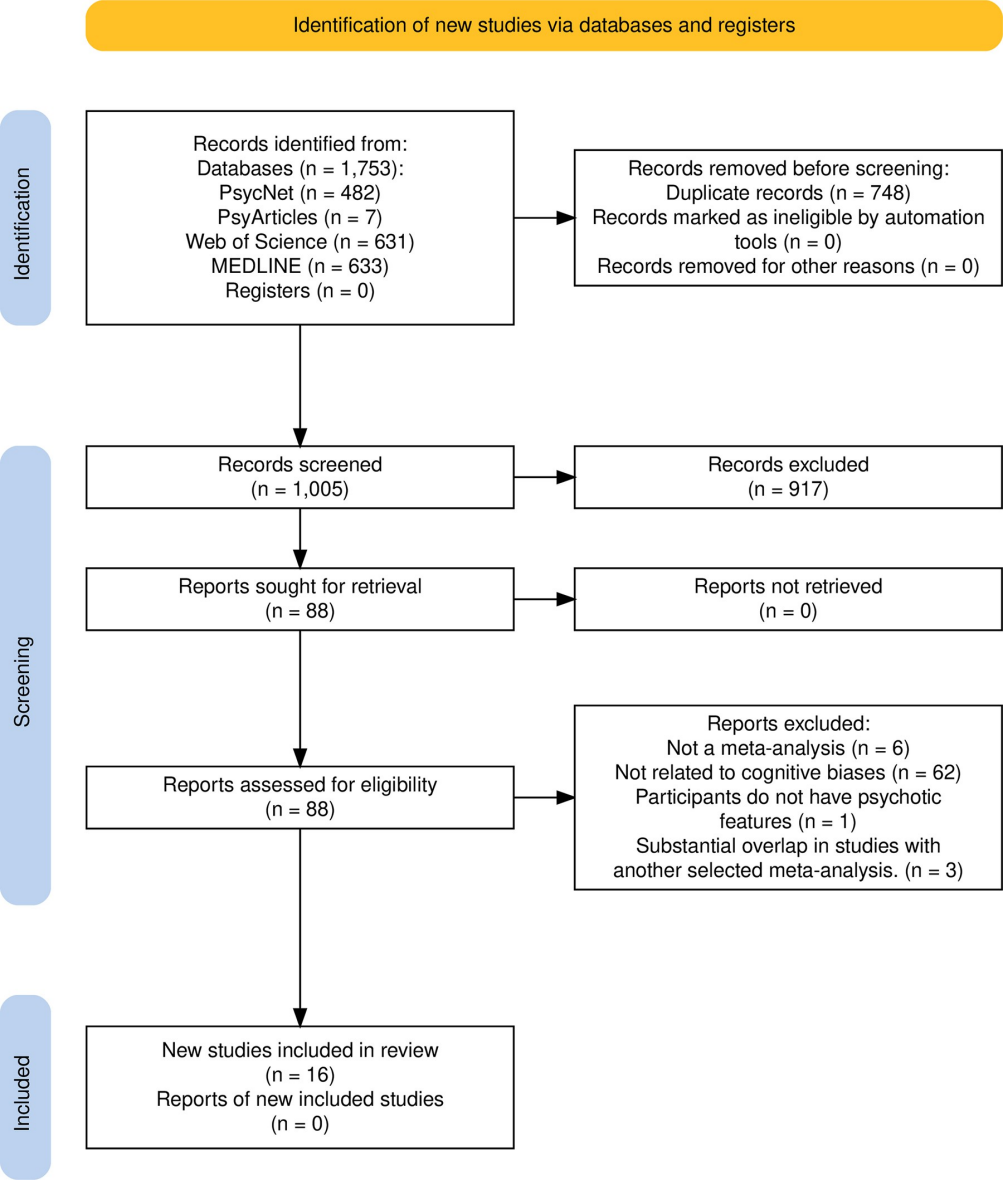
PRISMA 2020 Flow diagram of the articles’ selection process.

Overall, our search generated 1753 potential articles, from which 748 duplicates were removed, 848 articles were excluded based on titles and 69 were excluded based on abstract. Based on full text-read, 62 articles were excluded because they were not linked to interpretation or reasoning cognitive biases or were not reporting statistics specific to cognitive biases (e.g., some studies reported a general score of social cognition), six articles were excluded because they were not meta-analyses, one was removed because participants did not have any psychoticfeatures, three were removed because of substantial overlap in studies with another selected meta-analysis. Overall, 128 results were extracted and graded from the 16 meta-analyses remaining (Fig 1).

#### Nomenclature

In order to extract the results, a careful analysis of definitions, terms and measures used to measure the cognitive biases was conducted. This led to Table 1, which synthesizes the concepts measured in the meta-analyses under a single nomenclature, enabling us to aggregate together studies measuring the same concepts but that might have used different terminologies (Table 1).

**Table 1:** Cognitive bias classification and measures.

Category of biases or specific type of bias studied	Meta-analysis authors (year)	Bias name, as reported by authors	Description of the concept studied, as reported by authors	Measures used
**Cognitive biases**	Sauve et al. (2020) [25]	Cognitive biases	“Tendencies to systematically process, select and remember certain information”	4, 9, 11, 13, 15, 17, 21, 29, 30, 36 (possibility of being mistaken and reaction to hypothetical contradiction scores on the belief maintenance scale)
	Penney et al. (2022) [26]	Cognitive biases	“Maladaptive thinking styles common to psychosis”	9, 11, 13 (Scale or Factor Score), 14, 17—JTC/Lakes Task Confidence, 21, 23, 30, 36, Box Task [27], Probabilistic Reasoning Task [28]
**Interpretation biases**	Trotta et al. (2021) [29]	Interpretation biases	“Tendency to interpret emotionally ambiguous stimuli, situations, or events in a negative or positive manner”	1,3, 13 (Catastrophizing subscale),18, 20, 22, 23, 26, 28, 34, 35, 37
• **Attributional biases**	de Sousa et al. (2019) [30]	Attributional biases/style	“Causal inferences that individuals make about positive and negative social events”	3, 7, 19, 23
	Ventura et al. (2013) [31]	Attributional bias	“Inferring the causes of particular positive and negative events”	3, 7, 23
○ **Externalisation of cognitive events**	Brookwell et al. (2013) [32]	Externalizing bias	“Misattributions of internally generated cognitive events to external sources”	Source-Monitoring, Verbal Self-Monitoring and Signal Detection tasks
○ **Mix of attributional biases *(External-personal attribution*, *personalizing bias*, *internality attribution for negative events*, *externalizing bias)***	Murphy et al. (2018) [33]	Externalizing attributional biases	“Holding others responsible for negative events”	6 (External-Personal Attribution score for Negative Events), 7 (Expanded version, and Internality Attribution score for Negative Events), 8, 10 (External Attribution score for Negative Events), Interview transcripts rated using 12) (ore Attribution dataset, External-Personal Attribution score for Negative Events), 23 (Externalizing Bias Attribution score), 23 and 24 (Personalizing Bias Attribution score for Negative Events), 23 and 24 (External-Personal Attribution score for Negative Events), 25 (Speech samples coded using LACS, External-Personal Attribution score for Negative Events), 31 (Internality Attribution score for the Most Negative Event), 32 (external-personal attribution score for negative events)
○ **Self-serving bias**	Muller et al. (2021) [34]	Self-serving bias	“Tendency to attribute success to oneself, and failure to external factors”	7 (subtracting number of internal attributions for negative events from number of internal attributions for positive events), 23 (externalizing subscale), Investigators’ ratings based on utterances, others measures.
	Savla et al. (2013) [35]	Externalizing bias	“Tendency to overattribute positive rather than negative events to oneself”	23 (self-serving bias score)
	Livet et al. (2020) [36]	Externalizing bias	“Tendency to attribute positive events to the self and negative events to the external world”	5 (locus of control scale), 14 (external attribution bias subscale), 23 (self-serving bias subscale), 33
○ **Personalizing bias**	Savla et al. (2013) [35]	Personalizing bias	“Tendency to attribute negative events to others rather than to situational factors”	23 (personalizing bias subscore)
	Livet et al. (2020) [36]	Personalizing bias	“Tendency to attribute negative events to another person specifically”	23 (personalizing bias subscore)
○ **Hostility attribution bias**	Livet et al. (2020) [36]	Attention to threat	“Excessive perception of others’ behavior as threatening”	3 (hostility perception subscore), 14 (attention to threat subscore)
○ **Aberrant salience**	Livet et al. (2020) [36]	Aberrant salience	“Over-assignation of salient information to neutral or familiar stimuli”	2
**Reasoning biases**				
• **Belief inflexibility**	Zhu et al. (2018) [37]	Belief inflexibility	“Difficulty to re-evaluate a belief already formed”	9 (bias against disconfirmatory evidence score), 36 (Interview; possibility of being mistaken, reaction to hypothetical contradiction and alternative explanation scores on the belief maintenance scale)
	Livet et al. (2020) [36]	Belief inflexibility	“Difficulty in the capacity of reflecting and modifying our own beliefs in the light of reflection”	14 (belief inflexibility subscore)
○ **Bias against disconfirmatory evidence (BADE)**	McLean et al. (2017) [38]	Bias against disconfirmatory evidence (BADE)	“Failing to adequately re-evaluate an initial interpretation of events in the face of incre2ng evidence against that interpretation”	9
○ **Bias against confirmatory evidence (BACE)**	McLean et al. (2017) [38]	Bias against confirmatory Evidence (BACE)	“Failing to adequately up-rate the plausibility of the true interpretation des26e additional supporting evidence”	9 (bias against confirmatory Evidence score)
○ **Liberal acceptance**	McLean et al. (2017) [38]	Liberal acceptance	“Plausibility of absurd interpretations is overrated”	9 (liberal acceptance score)
○ **Jumping to conclusions and/or data-gathering bias**	So et al. (2016) [39]	Jumping to conclusion’s data-gathering bias	“Tendency to make decisions with certainty based on insufficient information”	11, 17, Word tasks, Name tasks, Survey tasks, or another salient version of the bead task.
	Dudley et al., (2016) [40]	Extreme responding bias	“Make decisions on the base of less evidence”	11, 17 or a salient version of the task
	Dudley et al., (2016) [40]	Draw to decision bias	“Make decisions on the basis of less evidence”	11, 17 or a salient version of the task
	Van Oosterhout et al., (2016) [41]	Data-gathering bias	“Tendency to gather less data or evidence than healthy controls in order to reach a decision or accept a hypothesis”	11, 17
	Ross et al., (2015) [42]	Jumping to conclusion bias	“To accept hypotheses on the b2s of less evidence”	11
	Livet et al., (2020) [36]	Jumping to Conclusions bias	“Make hasty decisions without considering alternative explanations”	14 (jumping to conclusion subscore)

Notes.: *Measures of cognitive biases*: 1—Abbreviated Trustworthiness task [43, 44]; 2—ASI, Aberrant Salience Inventory [45]; 3—AIHQ, Ambiguous Intentions Hostility Questionnaire [46]; 4—Ambiguous Test Descriptions [47]; 5—ANSIE Locus of Control scale, Adult Nowicki Strickland Internal External Locus of Control scale [48]; 6—ARAT, Attributional Style: Achievement and Relationships Attributions Task [49]; 7—ASQ, Attributional Style Questionnaire [50]; 8—ASQ-B, ASQ modified by Brunstein [51]; 9—BADE task, Bias Against Disconfirmatory Evidence task [52]); 10—BAI-R, Gudjonsson Blame Attribution Inventory-Revised [53]; 11—Beads task [54]; 12—CAVE, Content Analysis of Verbatim Explanations [55]; 13—CBQp, Cognitive Biases Questionnaire for psychosis [56]; 14—DACOBS, Davos Assessment of Cognitive Biases Scale [57]; 15—EoE assessment, Explanations of Experiences assessment [58]; 16—Expended-ASQ, Expended- Attributional Style Questionnaire [59]; 17—Fish task [60]; 18—Gesture-Interpretation task [61]; 19—IbT, Intentionality bias Test [62]; 20—Identification of Intention Vignettes [63]; 21—Illusion of Controls task [64]; 22—Interpretation of Ambiguous Laughter [65]; 23—IPSAQ, Internal, Personal and Situational Attributions Questionnaire [66]; 24—IPSAQ-R, Internal, Personal and Situational Attributions Questionnaire-Revised [67]; 25 –LACS, Leeds Attributional Coding System [68]; 26—Letter-String Discrimination [69]; 27—PIT, Pragmatic Inference Task [70]; 28—Perception of Criminal Intent Vignettes [71]; 29—Recognition task [72, 73]; 30—Representativeness task [74]; 31—SDEI, Significant Daily Events Interview [75]; 32—SAQ, Social Attributions Questionnaire [76]; 33—SCSQ, Social Cognition and Screening Questionnaire [77]; 34—SRT, Similarity-Ratings task [72, 78]; 35—SST, Scrambled-Sentences task [79, 80]; 36—MADS, Maudsley Assessment of Delusion Schedule [81]; 37—VR, Virtual-Reality questionnaire [82].

### Evidence for cognitive biases studied in relation to psychotic characteristics

**Fig 2:**
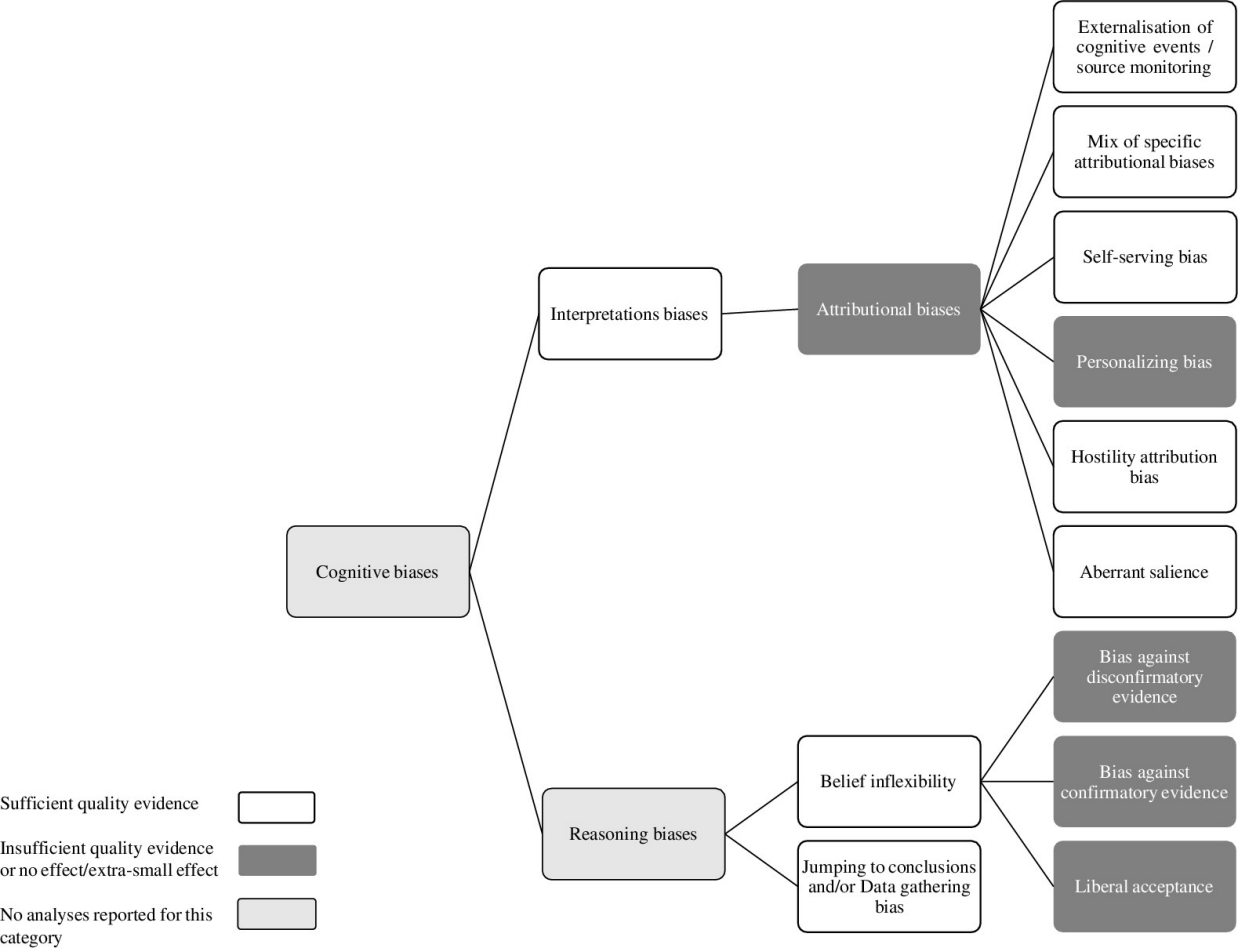
Classification of the cognitive biases studied.

The full results of the GRADE analysis are presented in supplementary material (S3 Table). Reasoning and interpretation biases can be broken down into smaller concepts, with specific biases studied. In Fig 2, we have attempted to synthesize the cognitive biases studied in meta-analyses on these two categories (reasoning and interpretation biases). As can be seen, attributional biases are a subtype of interpretation biases, and can be broken down into externalisation of cognitive events, or source monitoring, (with their valence–positive, neutral or negative), a mix of specific attributional biases, self-serving bias, personalizing bias, hostility attribution bias, and aberrant salience. Some authors also studied specific attributional biases regrouped together. Reasoning biases hold two larger categories, jumping to conclusions (JTC) and/or data gathering bias, and belief inflexibility being broken down into bias against disconfirmatory evidence (BADE), bias against confirmatory evidence (BACE), and liberal acceptance (LA) (Fig 2).

#### Interpretation biases

One meta-analysis [29] addressed different interpretation biases together. Large to very-large effect sizes were found for individuals with clinical and subclinical paranoia versus controls, with moderate quality evidence. Small to small-medium effect sizes were found for the correlation between severity of paranoid symptoms in clinical and non-clinical populations, with moderate to moderate-high-quality evidence (overall). Sample sizes were acceptable, confounding variables were addressed. Half of the analyses had consistent results and the other half received the point for the verification and absence of publication bias.

Subgroup comparisons of individuals with paranoia versus without paranoia (in clinical or non-clinical populations only) and association of interpretation biases with paranoia severity showed respectively small-medium and very large effect sizes with poor to poor-to-moderate quality evidence, mostly because results were not precise, samples sizes were too small, publication biases were not reported and results were not consistent across studies.

*Attributional biases*. Two meta-analyses [30, 31] addressed attributional biases in general, without differentiating them. The effect sizes ranged from no-effect or very small effect to medium-large effects for the association of attributional biases with disorganization, thought disorder, alogia [30] and reality distortion [31]. These studies had overall poor-quality evidence since all of them were imprecise, had small sample sizes and publication bias was not reported. Most of the results were also inconsistent and confounding variables were not addressed.

One meta-analysis [33] addressed a mix of four specific attributional biases (external-personal attribution, personalizing bias, internal attribution for negative Events, externalizing bias) in the objective to evaluate the tendency to hold others responsible for negative events. A medium effect size was found for the comparison between individuals with psychosis and persecutory delusions versus healthy controls, with moderate quality evidence. A small effect size was found for the association between attributional biases and paranoia severity in psychosis, with moderate quality evidence. These analyses had acceptable sample sizes, addressed confounding variables and publication biases were reported and absent.

Other comparisons (individuals with psychosis and persecutory delusions versus individuals with depression; individuals with psychosis and persecutory delusions versus individuals with psychosis without persecutory delusions) showed small-medium to very large effect sizes but the quality of evidence was poor to moderate. The results were imprecise, inconsistent and sample sizes were too small.

*Externalisation of cognitive events*. Only one meta-analysis [32] addressed attributional biases related to the externalization of cognitive events. A medium-large effect size was found for clinical populations with hallucinations and/or non-clinical hallucinations-prone populations compared to clinical populations without hallucinations and/or non-clinical populations not prone to hallucinations, with moderate-high-quality evidence. Results were consistent, sample size was acceptable, confounding variables were considered and publication bias was reported and absent. Sub-analyses by population groups (clinical only or subclinical only) showed medium-large to large effect sizes, but the quality evidence was poor to moderate. Results were not precise, sample sizes were too small, publication bias was present or not reported. Sub-analyses by stimulus valence (positive, negative or neutral) showed small to medium-large to large effect sizes, but the quality evidence was also poor. Results were not precise, inconsistent, sample sizes were too small and publication biases were not reported.

*Self-serving bias/externalizing bias*. Two meta-analyses specifically addressed self-serving biases [34, 36]. When comparing individuals with persecutory delusions to individuals with remitted persecutory delusions [34], there was a small effect size with moderate-high-quality evidence. A small-medium effect size was found for people with persecutory delusions when compared to non-clinical controls, with moderate quality evidence [34], and a very large effect size for schizophrenia spectrum disorders versus major depression, with moderate quality evidence [34]. Confounding variables were considered, publication biases were verified and absent, most of the sample sizes were acceptable or good. Moreover, comparisons between persecutory delusions versus remitted persecutory delusions showed precise and consistent results.

Comparison between individuals with schizophrenia and controls showed no effect or very-small effect size, with moderate quality evidence [34]. Sample size was good, confounding variables were considered, publication bias was verified and absent. However, results were not precise and were inconsistent. Other comparisons between groups (persecutory delusions versus schizophrenia spectrum disorders without persecutory delusions; persecutory delusions versus remitted persecutory delusion) [34] showed medium-large to large effect sizes, but the quality evidence was poor-to-moderate. The association of self-serving bias with measures of positive psychotic-like experiences in healthy samples and in ultra-high-risk samples, in healthy samples only or in ultra-high-risk samples only, or with measures of negative psychotic-like experiences in healthy samples only [36] showed medium-large effect sizes, and the quality of evidence was poor to poor-to-moderate. Results were inconsistent, confounding variables were not considered, and most of the results were imprecise.

Regarding the type of measures used for the self-serving bias, a large effect size was found for persecutory delusions compared to non-clinical controls, but only when measures other than the Attributional Style Questionnaire (ASQ) [50], and the Internal Personal and Situational Attribution Questionnaire (IPSAQ) [66] and investigators ratings were used [34]. Quality evidence was moderate because sample size was acceptable, confounding variables were considered and publication bias was verified and absent. However, results were not precise and were inconsistent.

Analyses using ASQ, IPSAQ or investigators ratings for the same group comparisons showed no effect or very small effect sizes, and/or did not have good enough quality evidence to conclude about the results [34]. A medium-large effect size was found between persecutory delusion versus non-clinical controls when measured with ASQ with poor to moderate quality evidence. Results were not precise, inconsistent et publication bias was present. When measured with IPSAQ, results showed no effect or very small effect size, with moderate quality evidence. Sample size was good, confounding variables were considered and publication bias was verified and absent. However, results were not precise and were inconsistent. When measured with investigators ratings based on utterances, results showed no effect or very small effect sizes, with poor quality evidence. Results were not precise, inconsistent, sample size was too small and publication bias was present.

*Personalizing bias*. Two meta-analyses [35, 36] specifically addressed the personalizing bias. Quality evidence for these results was insufficient to conclude on the effect. For instance, the comparison of individuals with schizophrenia versus controls [35] showed no effect or very-small effect sizes with poor quality evidence based on the results being imprecise, inconsistent, sample size too small and publication bias not reported.

Effect sizes varied between no effect or very small to large for the association between personalizing bias and measures of positive-psychotic-like experiences in healthy samples and ultra-high risk samples; in healthy samples only; and in ultra-high-risk samples only [36]. The reported results showed poor quality evidence with confounding variables not reported, and for most, the results were imprecise and the publication bias was not reported.

*Hostility attribution bias*. One meta-analysis [36] specifically addressed the hostility attribution bias. A medium effect size was found for the association with measures of negative psychotic-like experiences in healthy samples only, with moderate quality evidence. Results were precise, consistent and sample size was good. Other analyses (association with measures of positive psychotic-like experiences in all studies, in healthy samples only and in ultra-high-risk samples only) showed small-medium to very large effect size, but quality evidence was poor-to-moderate since confounding variables were not verified. Most of the results were inconsistent, and publication biases were not reported for most of the analyses.

*Aberrant salience*. One meta-analysis [36] addressed the Aberrant Salience bias. Association with positive psychotic-like experiences in healthy samples showed a very-large effect size, with moderate quality evidence. Results were precise, sample size was good and publication bias was verified and absent. Association with negative psychotic-like experiences in healthy samples showed a small effect size, with poor quality evidence. Results were not precise, inconsistent, confounding variables and publication bias were not reported.

#### Reasoning biases

*Belief inflexibility*. Two meta-analyses [36, 37] addressed belief inflexibility. A medium effect size was found for the relationship between belief inflexibility and global delusion severity in patients with psychotic disorders (patients with or without delusions), and a medium-large effect size was found for the relationship between belief inflexibility and delusional conviction in patients with psychotic disorders (patients with delusions only) [37], with moderate-high-quality evidence. Results were consistent, sample sizes were acceptable, confounding variables were considered and publication biases were verified and absent.

With moderate quality evidence, there was a medium effect size for the relationship between belief inflexibility and global delusion severity in patients with psychotic disorders (only participants with active delusions), a small effect size for the relationship between belief inflexibility and delusional preoccupation in patients with psychotic disorders (patients with delusions only) and a small effect size for the relationship between belief inflexibility and delusional distress in patients with psychotic disorders (patients with delusions only) [37]. Results were consistent, sample sizes were acceptable and confounding variables were considered.

When looking at analogue studies [36], the association of belief inflexibility with measures of positive psychotic-like experiences showed small effect sizes with poor quality evidence. The same effect size and quality evidence was shown for the healthy sample subgroup.

The association with measures of negative psychotic-like experiences in healthy samples only [36] also showed small effect sizes with poor quality evidence. Results were imprecise, inconsistent, and publication bias and confounding variables were not reported.

Regarding measures used for belief inflexibility, subgroup analyses for specific tasks showed no effect or very small to large effect sizes for interview-based measures and small-medium effect sizes for Bias against disconfirmatory evidence tasks (BADE-task) [83], with quality evidence from poor to poor-to-moderate [37]. Results were not precise, inconsistent, sample sizes were too small for half of the analyses and publication biases were not reported.

*Bias against disconfirmatory evidence*. One meta-analysis [38] specifically addressed the Bias Against Disconfirmatory evidence. Comparisons (schizophrenia with current delusions compared to healthy controls; schizophrenia with current delusions compared to schizophrenia without current delusions, schizophrenia without current delusions vs healthy controls; schizophrenia with current delusions compared to other psychiatric illnesses without current delusions) showed small-medium to medium-large effect sizes, with poor to poor-to-moderate quality evidence. Results were imprecise, sample sizes were too small and confounding variables were not considered. Half of the analyses did not report publication bias.

*Bias against confirmatory evidence*. One meta-analysis [38] specifically addressed the bias against confirmatory evidence. Comparisons (schizophrenia with current delusions versus healthy controls; schizophrenia with current delusions versus schizophrenia without current delusions; schizophrenia without current delusions versus healthy controls; schizophrenia with current delusions compared to other psychiatric illnesses without current delusions) showed small to medium effect sizes, but the quality evidence was poor. Results were imprecise, with small sample sizes, and publication bias and confounding variables were not reported.

*Liberal acceptance*. One meta-analysis [38] addressed the liberal acceptance bias. All of the analyses (comparisons between schizophrenia with current delusions vs healthy controls; schizophrenia with current delusions vs schizophrenia without current delusions; schizophrenia without current delusions vs healthy controls; and schizophrenia with current delusions vs other psychiatric illnesses without current delusions) had small-medium to large effect sizes, with poor quality evidence. Results were not precise, sample sizes were too small, confounding variables and publication biases were not reported.

*Jumping to conclusions (JTC) and/or data gathering bias*. Five meta-analyses [36, 38–40, 42] addressed the Jumping to conclusions and/or Data gathering bias. A medium-large effect size was found for the comparison between individuals with psychosis versus individuals with other mental health problems [40], with moderate-high-quality evidence. The results were precise, the sample size was moderate, confounding variables were considered, and the publication bias was verified. However, if only extreme responding was considered, the same comparison between individuals with psychosis versus individuals with other mental health problems for extreme responding showed large effect size [40], with poor quality evidence. The results were imprecise, the sample size was small, the confounding variable was considered and the publication bias was not verified.

A small effect size was found for the association of JTC with Peters et al’s. Delusions Inventory [84] scores overall (i.e. in the general population, those with current delusions, with previous delusions, with anxiety or depression, who are at risk of developing psychosis, with obsessional-compulsive disorder, and those in a new religious movement, grouped together), with moderate-high-quality evidence [42]. Samples sizes were good or acceptable, confounding variables were considered, publication bias was verified and absent. Results from the comparisons between individuals with psychosis versus individuals with other mental health problems were precise but inconsistent, whereas results for the association with Peters et al’s. Delusions Inventory scores, overall, were consistent but not precise. However, subgroup analyses (general population, current delusions, previous delusions, anxiety or depression, at-risk, obsessive-compulsive disorder, new religious movement) for the association between JTC and the Peters et al.’s Delusions Inventory showed no effect or extra-small to medium effect sizes, but the quality evidence was poor to poor-to-moderate. Most results were not precise and inconsistent, sample sizes were too small for most analyses and publication bias was not reported [42].

A medium effect size was found for the comparison between individuals with psychosis versus healthy individuals, and a large effect size was found for the same comparison when only extreme responding was considered, with moderate quality evidence [40]. Both sample sizes were large, confounding variables were considered and publication bias was verified. A medium-large effect size was found for the comparison between individuals with a psychotic disorder versus controls, with a moderate quality evidence [39]. The sample size was big, confounding variables were considered and publication bias was verified and absent.

A medium-large effect size was present when looking at the association between extreme responding in JTC and the presence or severity of delusions in psychosis with moderate quality evidence [40]. The results were consistent, the sample size was moderate and the publication bias was verified. However, when not addressing extreme responding, results for the association of JTC with delusion severity in people with psychosis and delusions showed no effect or very small effect size [40], with moderate quality evidence. The sample size was moderate, the confounding variables were considered and the publication bias was verified. A small-medium effect size was found for the comparison between schizophrenia with current delusions versus schizophrenia without current delusions [38] with moderate quality evidence. The results are consistent, the sample size is moderate and verification bias was verified. However, another meta- [40], with similar group comparison (comparison between individuals with psychosis and delusions versus individuals with psychosis without delusions) showed small effective size, with poor to moderate quality evidence. The results are imprecise, the sample size was small and the publication was not mentioned.

When looking at analogue studies, associations of JTC (measured with a self-report questionnaire) and measures of psychotic-like experiences (positive psychotic-like experiences in healthy and ultra-high-risk studies, and in healthy samples only, association with measures of negative psychotic-like experiences in healthy samples) [36] showed no effect to small effect sizes, but quality evidence was poor based on the results being not precise, inconsistent, confounding variables not considered and publication biases not reported.

Others group or subgroup comparisons studied by McLean et al. [38] (schizophrenia with current delusions versus healthy controls; other psychiatric illnesses with current delusions versus healthy controls; schizophrenia with current delusions versus other psychiatric illnesses with current delusions; schizophrenia with current delusions versus other psychiatric illnesses without current delusions; other psychiatric illnesses with current delusions versus other psychiatric illnesses without current delusions) showed small to large effect size, with poor to poor to moderate quality evidence. All of these analyses did not consider confounding variables and publication bias was not reported. Other group comparisons studied by So et al. [39] (patients with schizophrenia spectrum disorder versus healthy controls, patients with delusions versus controls) showed medium-large effect sizes, with quality evidence poor-to-moderate. The results were imprecise, inconsistent and the publication bias was not reported.

### Evidence for the effects of interventions on cognitive biases

In terms of the quality of the evidence for the effects of interventions on cognitive biases, psychological interventions in general [25] and Meta-Cognitive Training (MCT) in relation to the data-gathering bias more specifically [41] have been documented in meta-analyses. When analyzed together, the effects of psychological interventions MCT [9], and MCT adaptations, Maudsley review training programs (MRTP [85], MRTP in combination with the Interpretative Bias Modification (CBM-I; [86] or its adaptation, Thinking Well (TW) program [87], Reasoning Training (RT; [88], Cognitive Bias Correction (CBC; [89] and Cognitive Bias Modification (CBM; [90]) had small effect sizes on cognitive biases, with moderate-high overall quality evidence [25].

MCT had a small-medium effect size regarding the effect on jumping to conclusions (data-gathering bias) with poor overall quality evidence, since the results were not precise, inconsistent, the sample size was small, and publication bias was not considered [41]. MCT’s effect on cognitive biases as a large category, based on 19 studies (and 931 participants) reports a small effect size (g = 0.16) with moderate quality of evidence, based on good consistency, fairly large sample size and control for confounding variables, although when only RCTs were included (14 studies, 658 participants), the effect size became non-significant and the quality of the evidence also decreased to poor [26]. Furthermore, Penney et al. compared follow-up scores (less than 1 year, 10 studies, 658 participants) to both post-intervention and pre-intervention scores, revealing either small or very small effect sizes. Similarly, a very small effect size was observed between follow-up scores (more than 1 year, 3 studies, 328 participants) and post-intervention scores, but the quality of the evidence in these instances was poor or poor to moderate [26].

## Discussion

### Cognitive biases and psychotic characteristics

By grouping cognitive biases studied in meta-analyses under similar concepts, we can conclude that there is good evidence regarding the presence of the following alongside psychotic characteristics.

### Interpretations biases

Individuals with paranoia (clinical and subclinical regrouped) tend to show large interpretation biases when compared to controls. We also see small to small-medium correlations between the severity of paranoia and interpretation biases [29]. This concurs with existing theoretical models (e.g., Freeman).

*Attributional biases*. Two meta-analyses that studied multiple attributional biases [30, 31] grouped together found no effect to medium-large effect sizes, with insufficient quality evidence. However, Murphy et al. [33] showed that individuals with persecutory delusions had a greater tendency for a mix of specific attributional biases (external-personal attribution, personalizing bias, internal attribution for negative events, externalizing bias) (effect size was medium), with moderate quality of evidence. These contradictory results could be an artifact of studies looking at several biases together, given the amalgam of different concepts measures, and often small sample sizes.

*Externalisation of cognitive events*. Individuals with hallucinations and hallucination-prone individuals (clinical and non-clinical grouped together) have a greater tendency (effect size was medium-large) to misattribute internally generated cognitive events to external sources [32]. Although sub-analyses on stimuli valence were inconclusive and more quality studies are warranted, the current results seem congruent with the literature, whereby negative situations are often attributed to external causes to protect self-esteem [34].

*Self-serving bias/Externalizing bias*. Individuals with schizophrenia-spectrum disorders with persecutory delusions demonstrate a stronger self-serving bias than non-clinical controls, and then individuals with schizophrenia-spectrum disorders with remitted persecutory delusions (with small to small-medium effect sizes). There is no effect or a very-small effect size when comparing individuals with schizophrenia (with and without persecutory delusions) and controls, meaning the self-serving bias would be specific to persecutory delusions.

However, these results should be interpreted with caution, since sub-analyses reveal an absence of effect when using the ASQ [50] or IPSAQ [66], both widely used questionnaires for this bias. It is unclear at this moment if the other measures used are flawed, or measure the concept differently, or if the IPSAQ and ASQ have methodological issues, but studies using other measures report large effect sizes. Results also show that individuals with schizophrenia spectrum disorders have a greater tendency for this type of attribution compared to individuals with major depression, with a very large effect size. This is consistent with the literature on major depression, as depressed individuals lack the self-serving bias often found with controls [91].

*Personalizing bias*. The evidence was too poor to conclude on the relation between the personalizing bias and schizophrenia (with no effect or very small effect size) [35] and positive psychotic-like experiences in healthy samples and ultra-high risk samples (with no effect or very small effect sizes to large effect sizes) [36]. This bias was only measured with the IPSAQ. None of the meta-analyses in our review addressed the specific relation between the personalizing bias and paranoia or the severity of paranoia within clinical populations.

*Hostility attribution bias*. Hostility attribution bias was associated with negative-psychotic-like experiences in healthy samples (with a medium effect size), but quality evidence was poor-to-moderate [34]. Although these results are consistent with Rector and Beck’s theory [92] which suggests that negative symptoms of psychosis may be linked to social aversion and defeatist attitudes, the limited quality of evidence, the restricted sample (comprising only individuals with positive psychotic-like experiences in healthy or ultra-high-risk groups), and the lack of meta-analyses addressing this specific attributional bias in individuals with psychotic disorders collectively constrain our ability to confirm the role of hostility attribution bias in psychotic symptoms. The hostility attribution literature in schizophrenia and psychosis appears limited at this point, with a paucity of studies, mostly from the same team, suggesting links between hostility attribution bias and suspiciousness [93, 94].

*Aberrant salience bias*. Aberrant Salience bias was associated with positive psychotic-like experiences in healthy samples (with a very-large effect size), but with poor quality evidence. No meta-analysis in our review addressed this cognitive bias in individuals with psychotic disorders. This was particularly surprising as the aberrant salience literature has exploded, with over 200 studies in the past decade alone. For instance, a recent study [95] found a strong link between the aberrant salience bias and delusions, specifically ideas of reference. A meta-analysis on aberrant salience bias in psychosis is definitely warranted.

**Reasoning bias.** *Belief inflexibility bias*, *bias against disconfirmatory evidence*, *bias against confirmatory evidence*, *and liberal acceptance bias*. Belief inflexibility is associated with global delusion severity in patients with psychotic disorders and does not seem specific to delusions. It is also associated with delusion conviction, delusional preoccupation and delusional distress. Belief inflexibility biases show a medium effect size on global severity of delusion and a medium-large effect size delusional conviction, but only a small effect on delusional preoccupation and delusional distress suggesting that belief inflexibility biases bring more conviction in beliefs, but not much more distressed about them. These studies however did not look at the content of the delusions, which might further explain this result as paranoid delusions and grandiose delusions can trigger different levels of distress. Regarding studies on healthy and ultra-high-risk samples, the quality of the evidence is too poor at the moment to conclude.

As mentioned by Zhu and colleagues [37], the choice of measure can greatly influence the results here as some studies used items from the Maudsley Assessment of Delusions Schedule (MADS [81]), which overlap with measures of psychotic symptoms. and thus increase the effect size, particularly when compared to studies where belief inflexibility is measured with a task unrelated to the individual’s delusions (e.g., the BADE task [83]). As such, we found a large effect size between delusional conviction and MADS subscales but only a small-medium effect size between delusional conviction and BADE tasks, although the quality evidence was insufficient to clearly conclude about the effects of specific tasks on the results.

Regarding the Bias Against Disconfirmatory evidence [37, 38], insufficient overall quality evidence limits our ability to confirm or not the link with psychotic symptoms.

Overall quality evidence was also insufficient regarding the Bias against confirmatory evidence and the Liberal acceptance bias, both measured with scores derived from the BADE task. Methodologically sound studies using the BADE task, perhaps using emotion-triggering as well as neutral information, are warranted before we can conclude if this measure captures these reasoning biases well in individuals with psychotic characteristics.

*Jumping to conclusions (JTC) and/or data gathering bias*. Individuals with psychosis show a larger jumping to conclusions bias (Data-Gathering bias) than healthy individuals, both when comparing numbers of draws to decision and when considering only extreme answers (i.e. deciding after 1 or 2 beads), with medium to large effect sizes [40] and moderate to high quality evidence (Table 2). Individuals with psychosis also show a larger JTC (Data-Gathering) bias than individuals with other mental health problems, when comparing number of draws before making a decision, with a medium-large effect size [39, 40].

**Table 2:** Effect sizes of cognitive bias concepts with moderate to high-quality evidence.

Category of biases or specific type of bias studied	Meta-analysis authors (Year)	Outcome	Effect size	Overall quality evidence
Interpretation biases	Trotta et al. (2021) [29]	Individuals with clinical and subclinical paranoia versus controls (after one outlier within the clinical population subgroup was removed)	Large	Moderate
		Association with severity of paranoid symptoms in clinical and non-clinical studies	Small-medium	Moderate
		Association with severity of paranoid symptoms in clinical and non-clinical studies after two outliers’ studies in clinical subgroup was removed	Small	Moderate
• Attribution biases				
○ Externalisation of cognitive events	Brookwell et al. (2013) [32]	Clinicals with hallucinations and/or non-clinicals hallucinations prone versus clinicals without hallucinations and/or non-clinicals not prone	Medium-large	Moderate-high
		Clinicals with hallucinations and/or non-clinicals hallucinations prone versus clinicals without hallucinations and/or non-clinicals not prone, measured with source-monitoring task only	Medium	Moderate
○ Mix of attributional biases (external-personal attribution, personalizing bias, internality attribution for negative events, externalizing bias)	Murphy et al. (2018) [33]	Individuals with psychosis and persecutory delusions versus healthy controls	Medium	Moderate
		Association with paranoia severity in psychosis	Small	Moderate
○ Self-serving bias	Muller et al. (2021) [34]	Individuals with schizophrenia-spectrum disorders versus non-clinical controls	Small	Moderate
		Individuals with persecutory delusions versus non-clinical controls	Small-medium	Moderate
		Individuals with persecutory delusions versus individuals with remitted persecutory delusions	Small	Moderate-high
		Individuals with persecutory delusions versus non-clinical controls, measured with IPSAQ only	No effect or extra small	Moderate
		Individuals with persecutory delusions versus non-clinical controls, measured with others measures	Large	Moderate
		Individuals with schizophrenia spectrum disorders versus major depression	Very-large	Moderate
○ Hostility attribution bias	Livet et al. (2020) [36]	Association with measures of negative psychotic-like experiences in healthy samples only	Medium	Moderate
○ Aberrant salience	Livet et al. (2020) [36]	Association with positive psychotic-like experiences in healthy samples only	Very-Large	Moderate
Reasoning biases				
• Belief inflexibility	Zhu et al. (2018) [37]	Relationship between belief inflexibility and global delusion severity in patients with a psychotic disorder (patients with or without delusions)	Medium	Moderate-high
		Relationship between belief inflexibility and global delusion severity in patients with a psychotic disorder, participants with active delusions only	Medium	Moderate
		Relationship between belief inflexibility and delusional conviction in patients with a psychotic disorder, patients with delusions only	Medium-large	Moderate-high
		Relationship between belief inflexibility and delusional preoccupation in patients with a psychotic disorder, patients with delusions only	Small	Moderate
		Relationship between belief inflexibility and delusional distress in patients with a psychotic disorder, patients with delusions only	Small	Moderate
▪ Jumping to conclusions and/or data-gathering	Dudley et al. (2016) [40]	Individuals with psychosis versus healthy individuals (draw to decision bias)	Medium	Moderate
		Association with delusion severity in individuals with psychosis and delusions	No effect or extra small	Moderate
	McLean et al. (2017) [38]	Individuals with schizophrenia and current delusions versus individuals with schizophrenia without current delusions	Small-medium	Moderate
	Ross et al. (2015) [42]	Association with PDI scores, in overall (general population, individuals with current delusions, individuals with previous delusion, individuals with anxiety or depression, individuals at risk for psychosis, individuals with a obsessive-compulsive disorder and individuals in a new religious movement, grouped together)	Small	Moderate-high
	Dudley et al. (2016) [40]	Individuals with psychosis vs healthy individuals (extreme responding)	Large	Moderate

Notes.: IPSAQ, Internal, Personal and Situational Attributions Questionnaire [66]; PDI, Peters et al. Delusion Inventory [84]

Only extreme responding in JTC (i.e., deciding after a single or two beads/fish) is associated with the presence or severity of delusions in psychosis, with a medium-large effect size [40]. It is not clear however if extreme responding reveals more a lack of understanding of the task, a ‘need for closure’ (motivation to achieve absoluteness in judgements) [17], greater impulsivity or a stronger reasoning bias [40].

Comparisons between individuals with schizophrenia or a psychotic disorder with delusions versus those without delusions show a small effect size, with high quality evidence, which appears to support the idea that JTC might measure proneness to delusions but not necessarily delusions themselves [96].

When using the Peters et al’s Delusions Inventory [84] to assess delusional thinking, JTC biases were found in people from the general population, individuals with current delusions, individuals with previous delusions, individuals with anxiety or depressive disorders, individuals with ultra-high-risk for psychosis, individuals with obsessive-compulsive disorder and individuals in a new religious movement), with small effect sizes. There is no evidence that JTC, as measured with a self-report questionnaire, is related to positive- or negative-psychotic-like experiences in healthy and ultra-high-risk for psychosis individuals [36]. Validation studies on the Cognitive Bias Questionnaire [56] do suggest more important biases, including JTC bias, in people with psychosis when compared to the non-clinical controls but do not show convergent validity with JTC or other cognitive bias tasks. A meta-analysis that addresses JTC using self-report questionnaires in clinical populations is required.

### Effects of psychological interventions on cognitive biases

Regarding the effects of psychological interventions on cognitive biases, we found moderate-high-quality evidence and a small effect size when grouping together interventions targeting cognitive biases (Table 3). MCT was the intervention used in the majority of studies in Sauve et al.’s meta-analysis [25] and all of the studies in Penney et al’s meta-analysis [26]. When looking at the effects of MCT on the data gathering bias only, the quality of evidence is weaker, given that the results were not precise, not consistent, the sample size was small and publication bias was not considered. However, these results were part of a larger meta-analysis on the effects on MCT on cognition, which explains the small sample size in the sub analyses and why publication bias was not verified in this particular analysis. Overall, we find a small effect size on cognitive biases, with moderate quality of the evidence overall, but no effect and poor or poor to moderate quality of the evidence when including follow-up data or only high-quality studies. This might appear surprising as meta-analyses show that MCT improves psychotic symptoms (small-medium effect size), but cognitive biases that are theorized and targeted in the training to improve these symptoms, only show a small effect size in terms of improvements. Are we faced with a sensitivity-measure issue, whereby changes in biases are not detected by the measures used in the trials, or are other mediating variables at play? Future studies need to explore this question as the theoretical underpinnings of MCT could otherwise be questioned.

**Table 3:** Evidence for the effect of interventions targeting cognitive biases.

Cognitive bias(es) reported as by the Authors	Meta-analysis authors (year)	Type of intervention	Conclusion on effect	Overall quality evidence
Cognitive biases	Sauve et al. (2020) [25]	Effects of psychological interventions (MCT and MCT adaptations, MRTP, MRTP in combination with CBM-I or the TW program, RT, CBC and CBM)	Small	Moderate-high
		• Studies with high-risk bias only	Small-medium	Moderate
		• Studies with low-risk bias only	No effect or extra small	Poor to moderate
		• Studies with absence of active control conditions only	Small	Moderate
		• Studies with presence of active control conditions only	Small	Moderate
	Penney et al. (2022) [26]	MCT: individuals with schizophrenia spectrum and related psychotic disorders		
		• Pre versus post intervention	No effect or extra small	Moderate
		• Pre versus post intervention, RCTs only	No effect or extra small	Poor
		• Less than 1 year follow-up compared to the post-intervention scores	No effect or extra small	Poor to moderate
		• More than 1 year follow-up compared to the post-intervention scores	No effect or extra small	Poor to moderate
		• Less than 1 year follow-up compared to the pre-intervention scores)	Small	Poor
Data gathering biases	van Oosterhout et al. (2016) [41]	Effects of MCT	Small-medium	Poor

Notes.: CBC, Cognitive Bias Correction [89]; CBM, Cognitive Bias Modification [90, 100]; CBM-I, Cognitive Bias Modification for Interpretation [86]; MCT, Metacognitive Training [9]; MRTP, Maudsley Review Training Programme [85]; RT, Reasoning Training [88]; TW program, Thinking Well program [87]

It is also important to mention that no meta-analysis specifically addressing the effects of cognitive behaviour therapy for psychosis (CBTp) on cognitive biases was found in our review. CBTp is a cognitively oriented intervention that targets cognitive biases in order to modify beliefs underlying hallucinations and delusions [97]. CBTp is a well-recognized evidence-based treatment [98], considered effective in reducing psychotic (positive and negative) symptoms as well as depressive and social anxiety symptoms, and in improving functioning and mood [99].

### Relations between cognitive biases

While this umbrella-review investigated the associations between cognitive biases and psychotic characteristics, it does not allow us to extrapolate on the relationships between cognitive biases, as was theoretically hypothesized in a recent review [1]. Several cognitive models of psychopathology suggest roles for more than one cognitive bias in the maintenance of different disorders, with bidirectional effects. This phenomenon has been named the combined effect of cognitive biases hypothesis [101].

For instance, Broyd et al.’s [102] model on the formation and maintenance of delusional beliefs suggests that some cognitive biases (and other cognitive factors) would be related to the formation of delusions, whereas others would be more related to the maintenance of delusions. Cognitive factors such as JTC (which, according to the authors, would be mediated by the Liberal acceptance bias and by Salience) would interfere with the individual’s ability to process the information encountered using a “top down” process, i.e. by considering the information already known to interpret new stimuli. A study using a Bayesian mathematical model suggested that not using top-down processes prevents new information from being appropriately integrated [103]. Other cognitive vulnerabilities such as difficulties related to metamemory [104] (i.e. metacognitive knowledge and processes that necessitate memory [105], confirmation bias and BADE would prevent the person from invalidating their delusional beliefs. It would be relevant to further clarify which cognitive biases are linked to the formation and/or maintenance of delusions, since these could allow us to adjust cognitive interventions according to the specific phases of psychosis (e.g., prodromal/at-risk phase or residual phase).

### Limitations

Our umbrella-review is limited by the meta-analyses it included. Several reviews reported inconsistent results. This could be partly explained by the lack of consensus in the literature regarding the nomenclature, the classification and the measures used regarding cognitive biases. As such, cognitive bias names are used by different authors to designate different concepts. The term “externalizing bias” for instance is used by Brookwell et al. [32] to designate “*misattribution of internally generated cognitive events to an external source”*, whatever their valence (neutral, positive and negative), by Savla et al. [35] and Livet et al. [36] to designate “*attributing external causes to negative situations and/or attributing internal causes to positive situations”*, and by Murphy et al. [33] to designate a group of attributional biases (including the external-personal attribution score, the personalizing bias, the internality attribution score for negative events and the externalizing bias score). Several other small distinctions between concepts and nomenclature can be found across the meta-analyses used in our review. The plethora of different measures for the same concepts can also explain the important discrepancies in effect sizes across studies.

There are likely multiple studies on specific cognitive biases in psychosis or schizophrenia that were not described here because they were not included in a meta-analysis. For instance, we did not find meta-analyses on other specific interpretation or reasoning biases such as the catastrophizing bias, the dichotomous thinking bias or the emotional-based reasoning bias [56]. This could be explained by the paucity of individual studies on some of these biases in psychosis, as well as the few well-validated instruments available to measure them. However, there are some individual studies [106, 107] reporting results on catastrophizing bias, using the catastrophizing interview procedure [108], as well as studies using the catastrophizing scale of Peters and al.’s Cognitive Bias Questionnaire for psychosis [56]–they have yet to be included in a meta-analysis. We did not find meta-analyses regarding cognitive biases in people with substance-induced psychosis, nor meta-analyses looking at CBTp’s effects on cognitive biases. Furthermore, meta-analyses on cognitive biases and psychotic features measured longitudinally were absent from our search. Moreover, other cognitive biases (attention to threat, aberrant salience) were only reported in Livet et al. [36] within analogue studies involving individuals with psychotic experiences, and have not been reviewed in meta-analyses that include clinical populations. Finally, results specifically on the personalizing bias were also only reported in Livet et al. [36] in analogue studies, and in Savla et al. [35] as a sub analysis of a larger study on social cognition, making the conclusion we can draw from the results limited. It would be important to address the personalizing bias in a future meta-analysis, and to compare it with results linked to the self-serving bias (often called externalizing bias) and the hostility attribution bias to determine if the personalizing bias, or the hostility attribution bias is more associated with persecutory delusions than the self-serving bias.

### Strengths

An important strength of this umbrella-review is the use of the GRADE system to assess the quality of meta-analyses investigating relationships between cognitive biases and psychotic characteristics, including both healthy individuals and individuals with psychotic disorders. This enabled us to clearly identify the links with psychotic symptoms and comparisons between populations on biases supported by strong evidence, as well as document gaps in the literature and the need for future studies. This umbrella-review covered several specific biases, under two larger categories of biases, namely reasoning and interpretation biases. These biases are currently the most studied cognitive biases in schizophrenia, although a growing literature is developing on other cognitive biases such as memory and attention biases [109, 110]. Our umbrella-review was also quite thorough, carefully reporting sub-analyses as well as main analyses and considering several definitions and measures.

## Conclusion

Our umbrella-review highlights the need to conduct more and better studies in order to improve the quality evidence for certain research questions regarding cognitive biases and psychosis. As we can see in supplementary materials many meta-analyses lost quality evidence points (using the GRADE system) because the results were imprecise, or heterogeneous. Indeed, effect sizes are not constant across individual studies because of the variety of sample sizes used, or the small sample sizes, to assess the same biases.

This umbrella-review aimed to examine the quality of the evidence and importance of the association between psychotic characteristics and interpretation and reasoning cognitive biases. Associations between biases and psychotic characteristics had good quality evidence with medium to large effect sizes for interpretation biases when grouped together, as well as for the externalization of cognitive events, and self-serving bias, when people with clinical or subclinical symptoms were compared to control conditions. Similar effect sizes and quality evidence were found for belief inflexibility bias (when measured with MADS), and the jumping to conclusions bias when studied with data-gathering experimental methods (extreme score and number of draws to decision). The personalizing bias, the bias against disconfirmatory evidence, the bias against confirmatory evidence, and the liberal acceptance bias still need further quality research before we can conclude on their relation with psychotic characteristics. The hostility attribution bias and the aberrant salience bias need to be reviewed with clinical samples (and not just at-risk or non-clinical samples). High heterogeneity of effect sizes across studies negatively impacted the overall quality of most studies, and could reflect issues with the variety of measures used for the same concepts. Imprecise results also reflect the small or varying sample sizes involved in studying specific biases in several studies. Psychological interventions targeting cognitive biases appear to have a small effect on cognitive biases, which dissipate over time. New meta-analyses, on CBTp for instance, are necessary to conclude with sufficient evidence the effects of the interventions on the targeted cognitive biases.

In conclusion, this umbrella review gives caution toward taking for granted the quality and importance of the evidence supporting our cognitive models of psychosis, and the mediating role of cognitive biases in psychological treatments for psychosis. We have solid proof for some links and biases, but more rigorous studies and meta-analytical reviews are needed. A consensual nomenclature and selection of common measures for cognitive biases would definitely improve the quality of future systematic reviews.

## Supporting information

S1 Table Conclusions on effect sizes. Note. d = Cohen’s d, g = Hedges’g, R/RS = Pearson correlation coefficients, OR = Odds ratio.
(DOCX)

S2 Table Criteria for the quality assessment using the GRADE system. Note. If I2 and Q was not conclusive, Q was used for bigger sample size.
(DOCX)

S3 Table Description of included studies and their statistical and GRADE characteristics. Note. **Conclusions on effect size.** d/g: < 0.2 = No effect or very small; 0.2 to < 0.3 = Small; 0. to < 0.45 = Small-Medium; 0.45 to < 0.55 = Medium; 0.55 to < 0.75 = Medium-Large; 0.75 to < 1 = Large; > 1 = Very Large. R or RS: < 0.1 = No effect or very small; 0.1 to < 0.2 = Small; 0.2 to < 0.3 = Medium; 0.3 to < 0.4 = Medium-Large; 0.4 to < 0.5 = Large; > 0.5 = Very Large. OR: < 1 = No effect or very small; 1 to < 1.25 = Small; 1.25 to < 1.50 = Medium; 1.50 to < 2.50 = Medium-Large; 2.50 to < 10 = Large **Size of the sample.** < 500 = 0 point; 500 to < 1000 = 0.5 point, > 1000 = 1 point. **Precision of effects.** Large CIs > 0.25 in either direction = 0 point; Tight CIs < 0.25 in either direction = 1 point. **Homogeneity of effects across studies.** I2> 30% or Q is significant = 0 point; I2 < 30% or Q is not significant = 1 point. **Follow-up data.** Absence of follow-up data = 0 point; Presence of follow-up data (less than six months) = 0.5 point; Presence of follow-up data (six months or more) = 1 point. **Publication bias.** Not verified or not reported or verified and presence of publication bias = 0 point; Verified and absence of bias = 1 point. **Confounding factors.** No verified = 0 point; Verified = 1 point. **Overall Quality.** Total points for all elements of the GRADE system measured: < 1 = poor; 1 to < 2 = Poor to Moderate; 2 to < 3 = Moderate; 3 to < 4 = Moderate-High; 4 to 6 = High.
(DOCX)

S4 Table PRISMA 2020 checklist. (DOCX)

S5 Table List of all screened records and reasons of exclusion. (XLSX)
