# Supplementary material for: Reasoning and interpretation cognitive biases related to psychotic characteristics: An umbrella-review
Source: PLoS One. 2024 Dec 27;19(12):e0314965. doi: 10.1371/journal.pone.0314965 (PMC11676521; doi:10.1371/journal.pone.0314965)
Supplement: S1 Table — Note. d = Cohen’s d, g = Hedges’g, R/RS = Pearson correlation coefficients, OR = Odds ratio. (DOCX) [file pone.0314965.s001.docx]

**S1 Table. Conclusions on effect sizes.**

|  | **Effects sizes**  **(absolute values are used)** | **Conclusions** |
| --- | --- | --- |
| **d or g** | < 0.2 | No effect or very small |
|  | 0.2 to < 0.3 | Small |
|  | 0. to < 0.45 | Small-Medium |
|  | 0.45 to < 0.55 | Medium |
|  | 0.55 to < 0.75 | Medium-Large |
|  | 0.75 to < 1 | Large |
|  | > 1 | Very Large |
| **R ou RS** | < 0.1 | No effect or very small |
|  | 0.1 to < 0.2 | Small |
|  | 0.2 to < 0.3 | Medium |
|  | 0.3 to < 0.4 | Medium-Large |
|  | 0.4 to < 0.5 | Large |
|  | > 0.5 | Very Large |
| **OR** | < 1 | No effect or very small |
|  | 1 to < 1.25 | Small |
|  | 1.25 to < 1.50 | Medium |
|  | 1.50 to < 2.50 | Medium-Large |
|  | 2.50 to < 10 | Large |

Note. d = Cohen's d, g = Hedges’g, R/RS = Pearson correlation coefficients, OR = Odds ratio.
