# Supplementary material for: Reasoning and interpretation cognitive biases related to psychotic characteristics: An umbrella-review
Source: PLoS One. 2024 Dec 27;19(12):e0314965. doi: 10.1371/journal.pone.0314965 (PMC11676521; doi:10.1371/journal.pone.0314965)
Supplement: S2 Table — Note. If I2 and Q was not conclusive, Q was used for bigger sample size. (DOCX) [file pone.0314965.s002.docx]

**S2 Table.** **Criteria for the quality assessment using the GRADE system.**

| **Component** | **Criteria** | | **Points or Conclusion** |
| --- | --- | --- | --- |
| **Size of the sample** | < 500 | | 0 |
|  | 500 to < 1000 | | 0.5 |
|  | > 1000 | | 1 |
| **Precision of effects** | Large CIs > 0.25 in either direction | | 0 |
|  | Tight CIs < 0.25 in either direction | | 1 |
| **Homogeneity of effects across studies** | I2> 30% or Q is significant* | | 0 |
|  | I2 < 30% or Q is not significant* | | 1 |
| **Follow-up data** | Absence of follow-up data | | 0 |
|  | Presence of follow-up data (less than six months) | | 0.5 |
|  | Presence of follow-up data (six months or more) | | 1 |
| **Publication bias** | Not verified *or* not reported *or* verified and presence of publication bias | | 0 |
|  | Verified and absence of bias | | 1 |
| **Confounding factors** | No verified | | 0 |
|  | Verified | | 1 |
| **Overall Quality** | Total points for all elements of the GRADE system measured | < 1 | Poor |
|  |  | 1 to < 2 | Poor to Moderate |
|  |  | 2 to < 3 | Moderate |
|  |  | 3 to < 4 | Moderate-High |
|  |  | 4 to 6 | High |

Note. If I2 and Q was not conclusive, Q was used for bigger sample size
