# Supplementary material for: Reasoning and interpretation cognitive biases related to psychotic characteristics: An umbrella-review
Source: PLoS One. 2024 Dec 27;19(12):e0314965. doi: 10.1371/journal.pone.0314965 (PMC11676521; doi:10.1371/journal.pone.0314965)
Supplement: S3 Table — Note. Conclusions on effect size. d/g: < 0.2 = No effect or very small; 0.2 to < 0.3 = Small; 0. to < 0.45 = Small-Medium; 0.45 to < 0.55 = Medium; 0.55 to < 0.75 = Medium-Large; 0.75 to < 1 = Large; > 1 = Very Large. R or RS: < 0.1 = No effect or very small; 0.1 to < 0.2 = Small; 0.2 to < 0.3 = Medium; 0.3 to < 0.4 = Medium-Large; 0.4 to < 0.5 = Large; > 0.5 = Very Large. OR: < 1 = No effect or very small; 1 to < 1.25 = Small; 1.25 to < 1.50 = Medium; 1.50 to < 2.50 = Medium-Large; 2.50 to < 10 = Large Size of the sample. < 500 = 0 point; 500 to < 1000 = 0.5 point, > 1000 = 1 point. Precision of effects. Large CIs > 0.25 in either direction = 0 point; Tight CIs < 0.25 in either direction = 1 point. Homogeneity of effects across studies. I2> 30% or Q is significant = 0 point; I2 < 30% or Q is not significant = 1 point. Follow-up data. Absence of follow-up data = 0 point; Presence of follow-up data (less than six months) = 0.5 point; Presence of follow-up data (six months or more) = 1 point. Publication bias. Not verified or not reported or verified and presence of publication bias = 0 point; Verified and absence of bias = 1 point. Confounding factors. No verified = 0 point; Verified = 1 point. Overall Quality. Total points for all elements of the GRADE system measured: < 1 = poor; 1 to < 2 = Poor to Moderate; 2 to < 3 = Moderate; 3 to < 4 = Moderate-High; 4 to 6 = High. (DOCX) [file pone.0314965.s003.docx]

**S3 Table. Description of included studies and their statistical and GRADE characteristics.**

| **Category of biases or specific type of bias studied, as regrouped in the meta- review** | **Authors (year)** | **Cognitive bias and/or intervention, as named by the authors** | **Description of the outcome** | **Study design** | **Effect size** | | | | **Conclusion**  **on effect** | **Precision** | **Precision** | **Consistency** | | **Consistency** | **Number of studies (k) and number**  **of participants (N)** | | **Length of follow-up** | **Confounding variables verified** | **Publication bias** | **Total GRADE**  **score** | **Conclusion on effect size** | **Conclusion on overall quality** |
| --- | --- | --- | --- | --- | --- | --- | --- | --- | --- | --- | --- | --- | --- | --- | --- | --- | --- | --- | --- | --- | --- | --- |
|  |  |  |  |  | **d/g** | **R** | **RS** | **OR** |  | **95% Confidence Interval** | **(Yes/No)** | **Q** | **I² (%)** | **(Yes/No)** | **k** | **N** |  |  |  |  |  |  |
| **Cognitive biases** | Sauve et al. (2020) | Efficacy of psychological interventions targeting cognitive biases | Effect or efficacity on all studies, participants with schizophrenia spectrum disorders | Randomised controlled trials | 0,267 |  |  |  | Small | [1.127, 0.408] | No | N/A | 23,62 | Yes | 20 | 1085 | No follow-up reported | Yes | Verified, presence of  publication bias unprobable | 4 | Small | Moderate- High |
|  |  |  | Studies with high risk biais only | Randomised  controlled trials | 0,350 |  |  |  | Small-  Medium | [0.18, 0.53] | No | 6,95 | 0 | Yes | 8 | 1758 | No follow-up  reported | Yes | Not verified or not  reported | 3 | Small-  Medium | Moderate |
|  |  |  | Studies with low risk biais only | Randomised  controlled trials | 0,140 |  |  |  | No effect or  very small | [-0.07, 0.34] | No | 14,146 | 50,52 | No | 8 | 1157 | No follow-up  reported | Yes | Not verified or not  reported | 2 | No effect or  very small | Poor to  Moderate |
|  |  |  | Studies with presence of active control conditions only | Randomised  controlled trials | 0,270 |  |  |  | Small | [0.08, 0.47] | No | 10,986 | 8,97 | Yes | 11 | 433 | No follow-up  reported | Yes | Not verified or not  reported | 3 | Small | Moderate |
|  |  |  | Studies with absence of active control conditions only | Randomised  controlled trials | 0,200 |  |  |  | Small | [-0.04, 0.45] | No | 11,497 | 47,81 | Yes | 5 | 496 | No follow-up  reported | Yes | Not verified or not  reported | 3 | Small | Moderate |
|  | Penney et al. (2022) | Efficacy of MCT interventions targeting cognitive biases | individuals with schizophrenia spectrum and related psychotic disorders (Pre versus post intervention) | Comparative Not limited to Randomised  controlled trials | 0,160 |  |  |  | No effect or very small | [-0.02, 0.30] | No | 23,57 | 23,63 | Yes | 19 | 931 | No follow-up reported | Yes | Verified and presence of publication bias | 3 | No effect or very small | Moderate |
|  |  |  | individuals with schizophrenia spectrum and related psychotic disorders (Pre versus post intervention) | Comparative RCT only | 0,140 |  |  |  | No effect or very small | [-0.01, 0.31] | No | informa tion is missing | inform ation is missin g | information is missing | 14 | 658 | No follow-up reported | information is missing | information is missing | 0 | No effect or very small | Poor |
|  |  |  | individuals with schizophrenia spectrum and related psychotic disorders (Comparison of the follow-up (less than 1 year) to the post-intervention scores) | Comparative (follow up only) | 0,06 |  |  |  | No effect or very small | [-0.12, 0.24] | No | 14,59 | 38,33 | Yes | 10 | 658 | Follow-up less than one year | information is missing | information is missing | 2 | No effect or very small | Poor to Moderate |
|  |  |  | individuals with schizophrenia spectrum and related psychotic disorders (Comparison of the follow-up (more than 1 year) to the post-intervention  scores) | Comparative (follow up only) | -0,04 |  |  |  | No effect or very small | [-0.29, 0.21] | No | 1,27 | 0 | Yes | 3 | 328 | Follow-up more than one  year | information is missing | information is missing | 2 | No effect or very small | Poor to Moderate |
|  |  |  | individuals with schizophrenia spectrum and related psychotic disorders (comparison of the follow-up (less than 1 year) to the  pre-intervention scores) | Comparative (follow up only) | 0,2 |  |  |  | Small | [-0,02, 0,41] | No | 19,9 | 54,78 | No | 10 | 658 | Follow-up less than one year | information is missing | information is missing | 0 | Small | Poor |
| **Interpretation biases** | Trotta et al. (2021) | Interpretation biases | Individuals with clinical and subclinical paranoia versus controls | Comparative | 1.03 (Cohen's  d) |  |  |  | Very Large | [0.63, 1.43] | No | N/A | 82 | No | 11 | 705 | No follow-up  reported | Yes | Verified and no  publication bias | 3 | Very Large | Moderate |
|  |  |  | Individuals with clinical and subclinical paranoia versus controls, Likert scales only (if verified for Likert scales and experimental tasks, experimental tasks  include real life task) | Comparative | 1.3 (Cohen's d) |  |  |  | Very Large | [0,58, 2,02] | No | N/A | 89,8 | No | 5 | 508 | No follow-up reported | Yes | Not verified or not reported | 2 | Very Large | Poor to Moderate |
|  |  |  | Individuals with clinical and subclinical paranoia versus controls,  experimental tasks only (if verified for Likert scales and experimental tasks, experimental tasks include real life task) | Comparative | 0.78  (Cohen's d) |  |  |  | Large | [0,40, 1,17] | No | N/A | 55,07 | No | 6 | 368 | No follow-up reported | Yes | Not verified or not reported | 1 | Large | Poor |
|  |  |  | Individuals with clinical and subclinical paranoia versus controls, Likert scales only (if verified for Likert scales, experimental tasks and real life tasks,  experimental tasks exclude real life task) | Comparative | 1.3 (Cohen's d) |  |  |  | Very Large | [0,58, 2,02] | No | N/A | 89,8 | No | 5 | 508 | No follow-up reported | Yes | Not verified or not reported | 2 | Very Large | Poor to Moderate |
|  |  |  | Individuals with clinical and subclinical paranoia versus controls, experimental tasks only (if verified for Likert scales, experimental tasks and  real life tasks, experimental tasks exclude real life task) | Comparative | 0.91  (Cohen's d) |  |  |  | Large | [0,19, 1,64] | No | N/A | 63,9 | No | 2 | 160 | No follow-up reported | Yes | Not verified or not reported | 1 | Large | Poor |
|  |  |  | Individuals with clinical and subclinical paranoia versus controls, real life  tasks only (if verified for Likert scales, experimental tasks and real life tasks, experimental tasks exclude real life task) | Comparative | 0.72  (Cohen's d) |  |  |  | Medium- Large | [0,22, 1,21] | No | N/A | 58,1 | No | 4 | 208 | No follow-up reported | Yes | Not verified or not reported | 1 | Medium- Large | Poor |
|  |  |  | Individuals with clinical and subclinical paranoia versus controls (after one outlier within the clinical subgroup was removed) | Comparative | 0.775  (Cohen's d) |  |  |  | Large | [0.48, 1.07] | No | N/A | 37,3 | Yes | 10 | 623 | No follow-up reported | Yes | Not verified or not reported | 3 | Large | Moderate |
|  |  |  | Patients with psychosis versus unaffected control participants | Comparative | 1.014  (Cohen's d) |  |  |  | Very Large | [0.51, 1.52], p <  .001 | No | N/A | 83,9 | No | 7 | 216 | No follow-up  reported | Yes | Not verified or not  reported | 1 | Very Large | Poor |
|  |  |  | Non-clinical with subthreshold paranoia versus non-clinical witout paranoid  symptoms | Comparative | 1.065  (Cohen's d) |  |  |  | Very Large | [0.28, 1.85], p =  .008 | No | N/A | 83,6 | No | 4 | 245 | No follow-up  reported | Yes | Not verified or not  reported | 1 | Very Large | Poor |
|  |  |  | Association with severity of paranoid symptoms in clinical and non-clinical  (overall) | Correlational |  | 0,350 |  |  | Small-  Medium | [0.273, 0.426]; p  < .001 | Yes | N/A | 62,6 | No | 10 | 605 | No follow-up  reported | Yes | Verified, no publication  bias | 4 | Small-  Medium | Moderate-  High |
|  |  |  | Association with severity of paranoid symptoms in clinical and non-clinical  (overall), Likert scales only | Correlational |  | 0,260 |  |  | Medium | [0.16, 0.37] | No | N/A | 60 | No | 3 | 372 | No follow-up  reported | Yes | Not verified or not  reported | 1 | Medium | Poor |
|  |  |  | Association with severity of paranoid symptoms in clinical and non-clinical  (overall), experimental tasks only | Correlational |  | 0,450 |  |  | Large | [0.33, 0.56] | No | N/A | 45,4 | Yes | 7 | 492 | No follow-up  reported | Yes | Not verified or not  reported | 2 | Large | Poor to  Moderate |
|  |  |  | Association with severity of paranoid symptoms in clinical and non-clinical (overall) after two outliers studies in clinical population subgroup was  removed | Correlational |  | 0,270 |  |  | Small | [0.19, 0.36], p  value : significant | No | N/A | 13,1 | Yes | 8 | 561 | No follow-up reported | Yes | Not verified or not reported | 3 | Small | Moderate |
|  |  |  | Association with severity of paranoid symptoms in clinical | Correlational |  | 0,378 |  |  | Small-  Medium | [0.272, 0.484]; p  < .001 | No | N/A | 75,1 | No | 6 | 281 | No follow-up  reported | Yes | Not verified or not  reported | 1 | Small-  Medium | Poor |
|  |  |  | Association with severity of paranoid symptoms in non-clininal | Correlational |  | 0,319 |  |  | Small-  Medium | [0.21, 0.43], p <  .001 | No | N/A | 11,8 | Yes | 4 | 324 | No follow-up  reported | Yes | Not verified or not  reported | 2 | Small-  Medium | Poor to  Moderate |
| **Attributional biases** | de Sousa et al. (2019) | Attributional biases | Association with disorganisation, thought disorder and alogia in individuals  with schizophrenia spectrum disorders | Correlational |  | -0,143 |  |  | Small | [-0.347, 0.073] | No | N/A | 49,07 | No | 4 | 202 | No follow-up  reported | No | Not verified or not  reported | 0 | Small | Poor |
|  |  |  | Association with disorganisation in individuals with schizophrenia spectrum  disorders | Correlational |  | -0,307 |  |  | Medium-  Large | [-0.494, -0.092] | No | N/A | 0 | Yes | 2 | unclear | No follow-up  reported | No | Not verified or not  reported | 1 | Medium-  Large | Poor |
|  |  |  | Association with thought disorder in individuals with schizophrenia  spectrum disorders | Correlational |  | 0,060 |  |  | No effect or  very small | [-0.361, 0.461] | No | N/A | N/A | N/A | 1 | 23 | No follow-up  reported | No | Not verified or not  reported | 0 | No effect or  very small | Poor |
|  |  |  | Association with alogia in individuals with schizophrenia spectrum disorders | Correlational |  | 0,010 |  |  | No effect or  very small | [-0.204, 0.223] | No | N/A | N/A | N/A | 1 | 85 | No follow-up  reported | No | Not verified or not  reported | 0 | No effect or  very small | Poor |
|  | Ventura et al. (2013) | Attributional biases | Association with reality distortion | Correlational |  | -0,070 |  |  | No effect or  very small | Information is  missing | N/A | 25,87 | N/A | No | 6 | 250 | No follow-up  reported | Yes | Not verified or not  reported | 1 | No effect or  very small | Poor |
|  |  |  | Association with reality distortion, 2 studies dropped for homogenity | Correlational |  | -0,060 |  |  | No effect or  very small | Information is  missing | N/A | N/A | N/A | Yes | 4 | 185 | No follow-up  reported | Yes | Not verified or not  reported | 2 | No effect or  very small | Poor to  Moderate |
| **Externalisation of cognitive events** | Brookwell et al. (2013) | Externalising biais | Clinicals with hallucunations and/or non-clinicals hallucinations prone versus clinicals without hallucinations and/or non-clinicals not prone), source-monitoring task, verbal self-monitoring task and auditory signal  detection task | Comparative | 0,680 |  |  |  | Medium- Large | [0.48, 0.89] | No | 33,11 | 33,56 | Yes | Information is missing | 836 | No follow-up reported | Yes | Verified, no publication bias | 4 | Medium- Large | Moderate- High |
|  |  |  | Clinical with hallucinations versus clinical without hallucinations, source- monitoring task, verbal self-monitoring task and auditory signal detection  task | Comparative | 0,590 |  |  |  | Medium- Large | [0.46, 0.82] | No | 20,29 | 31,01 | Yes | 15 | 489 | No follow-up reported | Yes | Not verified or not reported | 2 | Medium- Large | Poor to Moderate |
|  |  |  | Analogue studies, hallucination-prone participants versus non hallucination- prone participants; association with hallucinations in non-clinicals, source- monitoring task, verbal self-monitoring task and auditory signal detection  task | Mix, comparative and correlationnal | 0,800 |  |  |  | Large | [0.54, 1.06] | No | 10,96 | 27,03 | Yes | 9 | 347 | No follow-up reported | Yes | Verified and presence of publication bias | 2 | Large | Poor to Moderate |
|  |  |  | Clinicals with hallucunations and/or non-clinicals hallucinations prone versus clinicals without hallucinations and/or non-clinicals not prone), auditory signal detection task only | Comparative | 0,870 |  |  |  | Large | [0.60, 1.14] | No | 10,99 | 27,26 | Yes | 9 | 350 | No follow-up reported | Yes | Verified and presence of publication bias | 2 | Large | Poor to Moderate |
|  |  |  | Clinicals with hallucunations and/or non-clinicals hallucinations prone  versus clinicals without hallucinations and/or non-clinicals not prone), source-monitoring task only | Comparative | 0,540 |  |  |  | Medium | [0.31, 0.76] | No | 17,89 | 28,52 | Yes | 13 | 500 | No follow-up reported | Yes | Not verified or not reported | 3 | Medium | Moderate |
|  |  |  | Clinicals with hallucunations and/or non-clinicals hallucinations prone versus clinicals without hallucinations and/or non-clinicals not prone), verbal  self-monitoring task only | Comparative | 0,380 |  |  |  | Small- Medium | [-0.17, 0.93] | No | N/A | N/A | N/A | 2 | 48 | No follow-up reported | Yes | Not verified or not reported | 1 | Small- Medium | Poor |
|  |  |  | Clinicals with hallucinations versus clinicals without hallucinations (sensivity analysis), source-monitoring task, verbal self-monitoring task and auditory  signal detection task | Comparative | 0,590 |  |  |  | Medium- Large | [0.46, 0.82] | No | 20,29 | 31,01 | Yes | Information is missing | 489 | No follow-up reported | No | Not verified or not reported | 1 | Medium- Large | Poor |
|  |  |  | Non-clinical halllucinations prone versus non clinical no hallucinations prone, source-monitoring task and and auditory signal detection task only | Comparative | 0,800 |  |  |  | Large | [0.54, 1.06] | No | 10,96 | 27,03 | Yes | Information is missing | 347 | No follow-up reported | No | Yes | 2 | Large | Poor to Moderate |
|  |  | Externalising biais (positive stimuli) | Clinicals with hallucunations and/or non-clinicals hallucinations prone versus clinicals without hallucinations and/or non-clinicals not prone), source-monitoring task and verbal self-monitoring task only, positive simuli | Comparative | 0,750 |  |  |  | Large | [0.45, 1.05] | No | N/A | N/A | N/A | Information is missing and not findable | Information is missing and not findable | No follow-up reported | Yes | Not verified or not reported | 1 | Large | Poor |
|  |  | Externalising biais (negative stimuli) | Clinicals with hallucunations and/or non-clinicals hallucinations prone versus clinicals without hallucinations and/or non-clinicals not prone), source-monitoring task and verbal self-monitoring task only, negative stimuli | Comparative | 0,620 |  |  |  | Medium- Large | [0.32, 0.91] | No | N/A | N/A | N/A | Information is missing and not findable | Information is missing and not findable | No follow-up reported | Yes | Not verified or not reported | 1 | Medium- Large | Poor |
|  |  | Externalising biais (neutral stimuli) | Clinicals with hallucunations and/or non-clinicals hallucinations prone versus clinicals without hallucinations and/or non-clinicals not hallucinations prone), source-monitoring task and verbal self-monitoring task  only, neutral stimuli | Comparative | 0,620 |  |  |  | Medium- Large | [0.32, 0.91] | No | N/A | N/A | N/A | Information is missing and not findable | Information is missing and not findable | No follow-up reported | Yes | Not verified or not reported | 1 | Medium- Large | Poor |
| **Mix of specific attributional biases** | Murphy et al. (2018) | External attribution bias | Individuals with psychosis and persecutory delusions versus healthy controls | Comparative | 0,480 |  |  |  | Medium | [0.23, 0.73] | No | N/A | 80 | No | 27 | 1442 | No follow-up  reported | Yes | Verified, no publication  bias | 3 | Medium | Moderate |
|  |  |  | Individuals with psychosis and persecutory delusions versus individuals with  depression | Comparative | 1,060 |  |  |  | Very Large | [0.48, 1.63] | No | N/A | 86 | No | 10 | 420 | No follow-up  reported | Yes | Verified, no publication  bias | 2 | Very Large | Poor to  Moderate |
|  |  |  | Individuals with psychosis and persecutory delusions versus individuals with  psychosis without persecutory delusions | Comparative | 0,400 |  |  |  | Small-  Medium | [0·12, 0.68] | No | N/A | 53 | No | 11 | 480 | No follow-up  reported | Yes | Verified, no publication  bias | 2 | Small-  Medium | Poor to  Moderate |
|  |  |  | Association with paranoia severity in psychosis | Correlational |  | 0,180 |  |  | Small | [0.08, 0.27] | No | N/A | 58 | No | 21 | 1128 | No follow-up  reported | Yes | Verified, no publication  bias | 3 | Small | Moderate |
| **Self-serving Bias/Externalizing Bias** | Livet et al. (2020) | External attribution bias | Association with measures of positive psychotic-like experiences in all studies  (healthy samples and ultra-high-risk samples) | Correlational |  |  | 0,350 |  | Medium-  Large | [0.25, 0.46] | No | 156.98 | 93 | No | 14 | 3958 | No follow-up  reported | No | Verified, no publication  bias | 2 | Medium-  Large | Poor to  Moderate |
|  |  |  | Association with measures of positive psychotic-like experiences in healthy  samples | Correlational |  |  | 0,360 |  | Medium-  Large | [0.26, 0.46] | No | 128.46 | 92,1 | No | 10 | 3682 | No follow-up  reported | No | Verified, no publication  bias | 2 | Medium-  Large | Poor to  Moderate |
|  |  |  | Association with measures of positive psychotic-like experiences in ultra-high-  risk samples | Correlational |  |  | 0,340 |  | Medium-  Large | [0.03, 0.64] | No | 28.26 | 89,9 | No | 4 | 148 | No follow-up  reported | No | Not verified or not  reported | 0 | Medium-  Large | Poor |
|  |  |  | Association with measures of negative psychotic-like experiences in healthy  samples | Correlational |  |  | 0,370 |  | Medium-  Large | [0.30, 0.45] | Yes | 12.5 | 67,9 | No | 4 | 1665 | No follow-up  reported | No | Not verified or not  reported | 2 | Medium-  Large | Poor to  Moderate |
|  | Muller et al. (2021) | Self-serving bias | Individuals with schizophrenia-spectrum disorders compared to non-clinical  contols | Comparative | 0,179 |  |  |  | No effect or  very small | [0.006, 0.35] | No | N/A | 85 | No | 56 | 5102 | No follow-up  reported | Yes | Verified, no publication  bias | 3 | No effect or  very small | Moderate |
|  |  |  | Individuals with persecutory delusion versus non-clinical controls | Comparative | 0,323 |  |  |  | Small-  Medium | [0.01, 0.63] | No | N/A | 85 | No | 25 | 1283 | No follow-up  reported | Yes | Verified, no publication  bias | 3 | Small-  Medium | Moderate |
|  |  |  | Individuals with persecutory delusion versus schizophrenia spectrum  disorders without persucutory delusions | Comparative | 0,575 |  |  |  | Medium-  Large | [0.0.23, 0.91] | No | N/A | 58 | No | 9 | 368 | No follow-up  reported | Yes | Verified, no publication  bias | 2 | Medium-  Large | Poor to  Moderate |
|  |  |  | Individuals with persecutory delusions versus remitted persecutory delusion | Comparative | 0,260 |  |  |  | Small | [-0.01, -0.537] | Yes | N/A | 0 | Yes | 5 | 210 | No follow-up  reported | Yes | Verified, no publication  bias | 4 | Small | Moderate-  High |
|  |  |  | Individuals with remitted persecutory delusions versus non-clinical controls | Comparative | -0,755 |  |  |  | Large | [-1.16, -0.34] | No | N/A | 51 | No | 5 | 219 | No follow-up  reported | Yes | Verified, no publication  bias | 2 | Large | Poor to  Moderate |
|  |  |  | Individuals with persecutory delusion versus non-clinical controls, mesured  with ASQ (attributionnal style questionnaire) only | Comparative | 0,711 |  |  |  | Medium-  Large | [0.13, 1.28] | No | N/A | 92 | No | 13 | 811 | No follow-up  reported | Yes | Verified, presence of  publication bias | 2 | Medium-  Large | Poor to  Moderate |
|  |  |  | Individuals with persecutory delusion versus non-clinical controls, mesured with IPSAQ (internal personal and situational attribution questoinnaire) only | Comparative | -0,060 |  |  |  | No effect or very small | [-0.24, 0.12] | No | N/A | 82 | No | 29 | 3581 | No follow-up reported | Yes | Verified, no publication bias | 3 | No effect or very small | Moderate |
|  |  |  | Individuals with persecutory delusion versus non-clinical controls, mesured  with investigators ratings based on utterances only | Comparative | -0,081 |  |  |  | No effect or  very small | [−0.40, 0.24] | No | N/A | 58 | No | 9 | 386 | No follow-up  reported | Yes | Verified, presence of  publication bias | 1 | No effect or  very small | Poor |
|  |  |  | Individuals with persecutory delusion versus non-clinical controls, mesured  with others mesures | Comparative | 0,782 |  |  |  | Large | [0.31, 1.24] | No | N/A | 74 | No | 5 | 503 | No follow-up  reported | Yes | Verified, no publication  bias | 3 | Large | Moderate |
|  |  |  | Individuals with schizophrenia spectrum disorders versus major depression | Comparative | 1,740 |  |  |  | Very Large | [1.14, 2.3] | No | N/A | 93 | No | 14 | 725 | No follow-up  reported | Yes | Verified, no publication  bias | 3 | Very Large | Moderate |

|  |  |  | Persecutory delusion versus non-clinical controls, predominant culture ; Asia  only | Comparative | 0,388 |  |  |  | Small-  Medium | [0.07, 0.71] | No | N/A | 84 | No | 11 | 1643 | No follow-up  reported | Yes | Verified, presence of  publication bias | 2 | Small-  Medium | Poor to  Moderate |
| --- | --- | --- | --- | --- | --- | --- | --- | --- | --- | --- | --- | --- | --- | --- | --- | --- | --- | --- | --- | --- | --- | --- |
|  |  |  | Persecutory delusion versus non-clinical controls, predominant culture ;  Western countries only | Comparative | 0,125 |  |  |  | No effect or  very small | [-0.07, 0.32] | No | N/A | 86 | No | 46 | 3459 | No follow-up  reported | Yes | Not verified or not  reported | 2 | No effect or  very small | Poor to  Moderate |
|  |  | Externalising bias | Individuals with schizophrenia versus controls | Comparative | -0,020 |  |  |  | No effect or  very small | [-0.40, 0.36] | No | 14,56 | N/A | No | 5 | 446 | No follow-up  reported | Yes | Not verified or not  reported | 1 | No effect or  very small | Poor |
| **Personalizing bias** | Livet et al. (2020) | Personalizing bias | Association with measures of positive psychotic-like experiences in all studies  (healthy samples and ultra-high-risk samples) | Correlational |  | 0,240 |  |  | Medium | [0.07, 0.41] | No | 48.54 | 87 | No | 6 | 1124 | No follow-up  reported | No | No publication bias | 2 | Medium | Poor to  Moderate |
|  |  |  | Association with measures of positive psychotic-like experiences in healthy  samples | Correlational |  | 0,030 |  |  | No effect or  very small | [-0.05, 0.10] | No | 0.00 | 0 | Yes | 2 | 731 | No follow-up  reported | No | Not verified or not  reported | 2 | No effect or  very small | Poor to  Moderate |
|  |  |  | Association with measures of positive psychotic-like experiences in ultra-high-  risk samples | correlational |  | 0,420 |  |  | Large | [0.34, 0.51] | Yes | 0.10 | 0,2 | Yes | 3 | 393 | No follow-up  reported | No | Not verified or not  reported | 2 | Large | Poor to  Moderate |
|  | Savla et al. (2013) | Personalizing bias | Individuals with schizophrenia versus controls | Comparative | -0,170 |  |  |  | No effect or  very small | [-0.72, 0.37] | No | 29,33 | N/A | No | 5 | 446 | No follow-up  reported | Yes | Not verified or not  reported | 1 | No effect or  very small | Poor |
| **Hostility attribution bias** | Livet et al. (2020) | Attention to threat | Association with measures of positive psychotic-like experiences in all studies  (healthy samples and ultra-high-risk samples) | Correlational |  |  | 0,380 |  | Medium-  Large | [0,32, 4.44] | No | 33,93 | 75,1 | No | 11 | 3154 | No follow-up  reported | No | Verified, no publication  bias | 2 | Medium-  Large | Poor to  Moderate |
|  |  |  | Association with measures of positive psychotic-like experiences in healthy  samples | Correlational |  |  | 0,340 |  | Medium-  Large | [0.27, 0.42] | Yes | 28,31 | 78,5 | No | 7 | 2736 | No follow-up  reported | No | Not verified or not  reported | 2 | Medium-  Large | Poor to  Moderate |
|  |  |  | Association with measures of positive psychotic-like experiences in ultra-high-  risk samples | Correlational |  |  | 0,470 |  | Large | [0.38, 0.55] | Yes | 1,8 | 19,2 | Yes | 4 | 163 | No follow-up  reported | No | Not verified or not  reported | 2 | Large | Poor to  Moderate |
|  |  |  | Association with measures of negative psychotic-like experiences in healthy  samples | Correlational |  |  | 0,280 |  | Medium | [0.23, 0.33] | Yes | 2,79 | 24,1 | Yes | 4 | 1665 | No follow-up  reported | No | Not verified or not  reported | 3 | Medium | Moderate |
| **Aberrant salience** | Livet et al. (2020) | Aberrant salience | Association with positive psychotic-like experiences in healthy samples | Information is  missing |  |  | 0,620 |  | Very Large | [0,56, 0,69] | Yes | 321,02 | 94,7 | No | 10 | 5165 | No follow-up  reported | No | Verified, no publication  bias | 3 | Very Large | Moderate |
|  |  |  | Association with negative psychotic-like experiences in healthy samples | Information is  missing |  |  | 0,180 |  | Small | [0,10, 0,26] | No | 29,71 | 83,1 | No | 8 | 3879 | No follow-up  reported | No | Not reported | 1 | Small | Poor |
| **Belief inflexibility** | Livet et al. (2020) | Belief inflexibility | Association with measures of positive psychotic-like experiences in all studies  (healthy samples and ultra-high-risk samples) | Correlational |  |  | 0,190 |  | Small | [0.11, 0.28] | No | 17,88 | 67,9 | No | 6 | 1905 | No follow-up  reported | No | Not verified or not  reported | 1 | Small | Poor |
|  |  |  | Association with measures of positive psychotic-like experiences in healthy  samples | Correlational |  |  | 0,180 |  | Small | [0.10, 0.26] | No | 16,69 | 67,6 | No | 5 | 1832 | No follow-up  reported | No | Not verified or not  reported | 1 | Small | Poor |
|  |  |  | Association with measures of negative psychotic-like experiences in healthy  samples | Correlational |  |  | 0,190 |  | Small | [0.03, 0.36] | No | 6,26 | 82,6 | No | 2 | 761 | No follow-up  reported | No | Not verified or not  reported | 1 | Small | Poor |
|  | Zhu et al. (2018) | Belief inflexibility | Relationship between belief inflexibility and global delusion severity in patients with a psychotic disorder (patients with or without delusions) | Mix, comparative and correlationnal | 0,452 |  |  |  | Medium | [0.303, 0.601] | No | N/A | 0 | Yes | 14 | 849 | No follow-up reported | Yes | Verified, no publication bias | 4 | Medium | Moderate- High |
|  |  |  | Relationship between belief inflexibility and global delusion severity in  patients with a psychotic disorder, only participants with active delusions | Information is  missing | 0,455 |  |  |  | Medium | [0.272, 0.638] | No | N/A | 0 | Yes | 13 | 579 | No follow-up  reported | Yes | Not verified or not  reported | 3 | Medium | Moderate |
|  |  |  | Relationship between belief inflexibility and delusional conviction in patients  with a psychotic disorders (patients with delusions only) | Information is  missing | 0,678 |  |  |  | Medium-  Large | [0.424, 0.932] | No | N/A | 32,44 | Yes | 9 | 565 | No follow-up  reported | Yes | Verified, no publication  bias | 4 | Medium-  Large | Moderate-  High |
|  |  |  | Relationship between belief inflexibility and delusional conviction in patients with a psychotic disorders (patients with delusions only), interview based-  only | Information is missing | 0,765 |  |  |  | Large | [0.462, 1.068] | No | N/A | N/A | information is missing | 5 | 550 | No follow-up reported | Yes | Not verified or not reported | 2 | Large | Poor to Moderate |
|  |  |  | Relationship between belief inflexibility and delusional conviction in patients with a psychotic disorders (patients with delusions only), BADE task only | Information is missing | 0,433 |  |  |  | Small- Medium | [0.02, 0.89] | No | N/A | N/A | information is missing | 4 | 110 | No follow-up reported | Yes | Not verified or not reported | 1 | Small- Medium | Poor |
|  |  |  | Relationship between belief inflexibility and delusional preoccupation in  patients with a psychotic disorders (patients with delusions only) | Information is  missing | 0,274 |  |  |  | Small | [0.098, 0.450] | No | N/A | 0 | Yes | 9 | 573 | No follow-up  reported | Yes | Verified, presence of  publication bias | 3 | Small | Moderate |
|  |  |  | Relationship between belief inflexibility and delusional preoccupation in  patients with a psychotic disorders (patients with delusions only), interview based-only | Information is missing | 0,252 |  |  |  | Small | [0.062, 0.443] | No | N/A | N/A | information is missing | 5 | 550 | No follow-up reported | Yes | Not verified or not reported | 2 | Small | Poor to Moderate |
|  |  |  | Relationship between belief inflexibility and delusional preoccupation in patients with a psychotic disorders (patients with delusions only), BADE task  only | Information is missing | 0,380 |  |  |  | Small- Medium | [0.171, 0.931] | No | N/A | N/A | information is missing | 4 | 110 | No follow-up reported | Yes | Not verified or not reported | 1 | Small- Medium | Poor |
|  |  |  | Relationship between belief inflexibility and delusional distress in patients  with a psychotic disorders (patients with delusions only) | Information is  missing | 0,200 |  |  |  | Small | [0.025, 0.376] | No | N/A | 0,103 | Yes | 9 | 576 | No follow-up  reported | Yes | Verified, presence of  publication bias | 3 | Small | Moderate |
|  |  |  | Relationship between belief inflexibility and delusional distress in patients  with a psychotic disorders (patients with delusions only), interview based- only | Information is missing | 0,157 |  |  |  | No effect or very small | [0.033, 0.346] | No | N/A | N/A | information is missing | 5 | 550 | No follow-up reported | Yes | Not verified or not reported | 2 | No effect or very small | Poor to Moderate |
|  |  |  | Relationship between belief inflexibility and delusional distress in patients  with a psychotic disorders (patients with delusions only), BADE task only | Information is  missing | 0,429 |  |  |  | Small-  Medium | [0.152, 1.1] | No | N/A | N/A | information is  missing | 4 | 110 | No follow-up  reported | Yes | Not verified or not  reported | 1 | Small-  Medium | Poor |
| **Bias Against Disconfirmatory Evidence (BADE)** | McLean et al. (2017) | Bias Against Disconfirmatory Evidence (BADE) | Individuals with schizophrenia and current delusions versus healthy controls | Comparative | 0,558 |  |  |  | Medium-  Large | [0.281, 0.834] | No | N/A | 38 | Yes | 7 | 369 | No follow-up  reported | No | Verified, no publication  bias | 2 | Medium-  Large | Poor to  Moderate |
|  |  |  | Individuals with schizophrenia and current delusions versus schizophrenia  without current delusions | Comparative | 0,308 |  |  |  | Small-  Medium | [0,017, 0.598] | No | N/A | 49,5 | No | 8 | 466 | No follow-up  reported | No | Verified, no publication  bias | 1 | Small-  Medium | Poor |
|  |  |  | Individuals with schizophrenia without current delusions versus healthy  controls | Comparative | 0,352 |  |  |  | Small-  Medium | [0.153, 0.551] | No | N/A | 0 | Yes | 7 | 455 | No follow-up  reported | No | Not verified or not  reported | 1 | Small-  Medium | Poor |
|  |  |  | Individuals with schizophrenia and current delusions versus other psychiatric  illnesses without current delusions | Comparative | 0,676 |  |  |  | Medium-  Large | [0.338, 1.014] | No | N/A | 20 | Yes | 4 | 211 | No follow-up  reported | No | Not verified or not  reported | 1 | Medium-  Large | Poor |
| **Bias Against Confirmatory Evidence (BACE)** | McLean et al. (2017) | Bias Against Confirmatory Evidence (BACE) | Individuals with schizophrenia and current delusions versus healthy controls | Comparative | 0,530 |  |  |  | Medium | [0.316, 0.744] | No | N/A | 0 | Yes | 7 | 369 | No follow-up  reported | No | Not verified or not  reported | 1 | Medium | Poor |
|  |  |  | Individuals with schizophrenia and current delusions versus individuals with  schizophrenia without current delusions | Comparative | 0,392 |  |  |  | Small-  Medium | [0.122, 0.535] | No | N/A | 0 | Yes | 7 | 426 | No follow-up  reported | No | Not verified or not  reported | 1 | Small-  Medium | Poor |
|  |  |  | Individuals with schizophrenia without current delusions versus healthy  controls | Comparative | 0,219 |  |  |  | Small | [-0.006, 0.444] | No | N/A | 19,4 | Yes | 7 | 455 | No follow-up  reported | No | Not verified or not  reported | 1 | Small | Poor |
|  |  |  | Individuals with schizophrenia and current delusions versus other psychiatric  illnesses without current delusions | Comparative | 0,481 |  |  |  | Medium | [0.185, 0.778] | No | N/A | 0 | Yes | 4 | 211 | No follow-up  reported | No | Not verified or not  reported | 1 | Medium | Poor |
| **Liberal acceptance (LA)** | McLean et al. (2017) | Liberal acceptance (LA) | Individuals with schizophrenia with current delusions versus healthy controls | Comparative | 0,779 |  |  |  | Large | [0.449, 1.108] | No | N/A | 50,6 | No | 6 | 338 | No follow-up  reported | No | Not verified or not  reported | 0 | Large | Poor |
|  |  |  | Individuals with schizophrenia with current delusions versus schizophrenia  without current delusions | Comparative | 0,382 |  |  |  | Small-  Medium | [0.148, 0.617] | No | N/A | 8,9 | Yes | 6 | 383 | No follow-up  reported | No | Not verified or not  reported | 1 | Small-  Medium | Poor |

|  |  |  | Individuals with schizophrenia without current delusions versus healthy  controls | Comparative | 0,477 |  |  |  | Medium | [0.170, 0.784] | No | N/A | 48,6 | No | 6 | 409 | No follow-up  reported | No | Not verified or not  reported | 0 | Medium | Poor |
| --- | --- | --- | --- | --- | --- | --- | --- | --- | --- | --- | --- | --- | --- | --- | --- | --- | --- | --- | --- | --- | --- | --- |
|  |  |  | Individuals with schizophrenia with current delusions versus other  psychiatric illnesses without current delusions | Comparative | 0,498 |  |  |  | Medium | [0.201, 0.794] | No | N/A | 0 | Yes | 4 | 211 | No follow-up  reported | No | Not verified or not  reported | 1 | Medium | Poor |
| **Jumping to conclusions and/or Data- gathering bias** | Dudley et al. (2016) | Draws to decision | Individuals with psychosis versus healthy individuals | Comparative | -0,520 |  |  |  | Medium | [-0.69, -0.36] | No | 93.67 | 66 | No | 33 | 1935 | No follow-up  reported | Yes | Verified, no publication  bias | 3 | Medium | Moderate |
|  |  |  | Individuals with psychosis versus individuals with other mental health  problems | Comparative | -0,580 |  |  |  | Medium-  Large | [-0.80, -0.35] | Yes | 22.42 | 46 | No | 13 | 667 | No follow-up  reported | Yes | Verified, no publication  bias | 4 | Medium-  Large | Moderate-  High |
|  |  |  | Individuals with psychosis and delusions versus individuals with psychosis  without delusions | Comparative | -0,290 |  |  |  | Small | [-0.48, -0.09] | No | 4.54 | 0 | Yes | 8 | 456 | No follow-up  reported | Yes | Not verified or not  reported | 2 | Small | Poor to  Moderate |
|  |  |  | Association with delusion severity in people with psychosis and delusions | Correlational | -0,180 |  |  |  | No effect or  very small | [-0.43,0.06] | No | 37.31 | 54 | No | 18 | 794 | No follow-up  reported | Yes | Verified, no publication  bias | 3 | No effect or  very small | Moderate |
|  |  | Jumping to conclusions (extreme responding) | Individuals with psychosis versus healthy individuals (extreme responding) | Comparative |  |  |  | 3,820 | Large | [2.69, 5.43] | No | N/A | 44 | No | 22 | 1431 | No follow-up  reported | Yes | Verified, no publication  bias | 3 | Large | Moderate |
|  |  |  | Individuals with psychosis versus individuals with other mental health  problems (extreme responding) | Comparative |  |  |  | 5,550 | Large | [2.32, 13.28] | No | N/A | 20 | Yes | 4 | 202 | No follow-up  reported | No | Not verified or not  reported | 1 | Large | Poor |
|  |  |  | Odd ratio, presence or severity of delusions in psychosis (extreme responding) | Comparative |  |  |  | 1,520 | Medium-  Large | [1.12, 2.05] | No | N/A | 13 | Yes | 14 | 770 | No follow-up  reported | No | Verified, no publication  bias | 3 | Medium-  Large | Moderate |
|  | Livet et al. (2020) | Jumping to conclusions | Association with measures of positive psychotic-like experiences in all studies  (healthy samples and ultra-high-risk samples) | Correlational |  |  | 0,100 |  | Small | [0.04, 0.17] | No | 16,62 | 67,7 | No | 8 | 2809 | No follow-up  reported | No | Not verified or not  reported | 1 | Small | Poor |
|  |  |  | Association with measures of positive psychotic-like experiences in healthy  samples | Correlational |  |  | 0,110 |  | Small | [0.03, 0.18] | No | 16,6 | 72,8 | No | 7 | 2736 | No follow-up  reported | No | Not verified or not  reported | 1 | Small | Poor |
|  |  |  | Association with measures of negative psychotic-like experiences in healthy  samples | Correlational |  |  | -0,090 |  | No effect or  very small | [-0.20, 0.02] | No | 16,43 | 80,5 | No | 4 | 1665 | No follow-up  reported | No | Not verified or not  reported | 1 | No effect or  very small | Poor |
|  | McLean et al. (2017) | Jumping to conclusions | Individuals with schizophrenia with current delusions versus healthy controls | Comparative | 0,707 |  |  |  | Medium-  Large | [0.514, 0.900] | No | N/A | 58,2 | No | 21 | 1131 | No follow-up  reported | No | Verified, possible slight  publication bias | 1 | Medium-  Large | Poor |
|  |  |  | Individuals with schizophrenia with current delusions versus schizophrenia  without current delusions | Comparative | 0,327 |  |  |  | Small-  Medium | [0.191, 0.463] | No | N/A | 0 | Yes | 20 | 834 | No follow-up  reported | No | Verified, no publication  bias | 3 | Small-  Medium | Moderate |
|  |  |  | Individuals with schizophrenia without current delusions versus healthy  controls | Comparative | 0,119 |  |  |  | No effect or  very small | [-0.171, 0.409] | No | N/A | 47,3 | No | 7 | 385 | No follow-up  reported | No | Not verified or not  reported | 0 | No effect or  very small | Poor |
|  |  |  | Other psychiatric illnesses with current delusions versus healthy controls | Comparative | 0,764 |  |  |  | Large | [0.437, 1.092] | No | N/A | 0 | Yes | 4 | 152 | No follow-up  reported | No | Not verified or not  reported | 1 | Large | Poor |
|  |  |  | Individuals with schizophrenia with current delusions versus other  psychiatric illnesses with current delusions | Comparative | 0,202 |  |  |  | Small | [-0.230, 0.633] | No | N/A | 0 | Yes | 2 | 86 | No follow-up  reported | No | Not verified or not  reported | 1 | Small | Poor |
|  |  |  | Individuals with schizophrenia with current delusions versus other  psychiatric illnesses without current delusions | Comparative | 0,839 |  |  |  | Large | [0.637, 1.042] | Yes | N/A | 0 | Yes | 10 | 409 | No follow-up  reported | No | Not verified or not  reported | 2 | Large | Poor to  Moderate |
|  |  |  | Other psychiatric illnesses with current delusions versus other psychiatric  illnesses without current delusions | Comparative | 0,611 |  |  |  | Medium-  Large | [0.219, 1.003] | No | N/A | 15,7 | Yes | 3 | 123 | No follow-up  reported | No | Not verified or not  reported | 1 | Medium-  Large | Poor |
|  | Ross et al. (2015) | Jumping to conclusions (draws to decision) | Association with Peters et al. Delusions Inventory scores, in overall (general population, current delusion, previous delusion, anxiety or depression, at risk, OCD and new religious mouvement) (draws to decision) | Correlational |  | -0,100 |  |  | Small | [-0.17, -0.03] | No | N/A | 33 | Yes | 38 | 237 | No follow-up reported | Yes | Verified, no publication bias | 4 | Small | Moderate- High |
|  |  |  | Association with Peters et al. Delusions Inventory scores in subgroup general  population (draws to decision) | Correlational |  | -0,100 |  |  | Small | [-0.18, -0.02] | No | N/A | 40 | No | 23 | 1754 | No follow-up  reported | Yes | Not verified or not  reported | 2 | Small | Poor to  Moderate |
|  |  |  | Association with Peters et al Delusions Inventory scores in subgroup current  delusions (draws to decision) | Correlational |  | -0,120 |  |  | Small | [-0.31, 0.07] | No | N/A | 40 | Yes | 8 | 262 | No follow-up  reported | Yes | Not verified or not  reported | 2 | Small | Poor to  Moderate |
|  |  |  | Association with Peters et al Delusions Inventory scores in subgroup previous  delusions (draws to decision) | Correlational |  | 0,050 |  |  | No effect or  very small | [-0.53, 0.63] | No | N/A | 78 | No | 2 | 54 | No follow-up  reported | Yes | N/A | 1 | No effect or  very small | Poor |
|  |  |  | Association with Peters et al Delusions Inventory scores in subgroup anxiety  or depression (draws to decision) | Correlational |  | -0,040 |  |  | No effect or  very small | [-0.28, 0.19] | No | N/A | 0 | Yes | 2 | 76 | No follow-up  reported | Yes | N/A | 2 | No effect or  very small | Poor to  Moderate |
|  |  |  | Association with Peters et al Delusions Inventory scores in subgroup at risk  (draws to decision) | Correlational |  | -0,150 |  |  | Small | [-0.55, 0.25] | No | N/A | N/A | N/A | 1 | 27 | No follow-up  reported | Yes | N/A | 1 | Small | Poor |
|  |  |  | Association with Peters et al. Delusions Inventory scores in subgroup OCD  (draws to decision) | Correlational |  | 0,000 |  |  | no effect or  extra small | [-0.36, 0.37] | N/A | N/A | N/A | N/A | 1 | 32 | No follow-up  reported | Yes | N/A | 1 | no effect or  extra small | Poor |
|  |  |  | Association with Peters et al Delusions Inventory scores in subgroup new  religious mouvement (draws to decision) | Correlational |  | 0,230 |  |  | Medium | [-0.60, 0.13] | Yes | N/A | N/A | N/A | 1 | 32 | No follow-up  reported | Yes | N/A | 2 | Medium | Poor to  Moderate |
|  | So et al. (2016) | Jumping to conclusions | Individuals with a psychotic disorder versus controls | Comparative | -0,601 |  |  |  | Medium-  Large | [-0.773, -0.428] | No | 176,46 | 77,9 | No | 39 | 2411 | No follow-up  reported | Yes | Verified, no publication  bias | 3 | Medium-  Large | Moderate |
|  |  |  | Patients with schizophrenia spectrum disorder (with and without delusions)  versus healthy controls | Comparative | -0,618 |  |  |  | Medium-  Large | [-0,817, -0,419] | No | N/A | N/A | N/A | 34 | unclear | No follow-up  reported | Yes | Not verified or not  reported | 2 | Medium-  Large | Poor to  Moderate |
|  |  |  | Patients with delusions versus controls | Comparative | -0,615 |  |  |  | Medium-  Large | [-0,857, -0,373] | No | N/A | N/A | N/A | 31 | unclear | No follow-up  reported | Yes | Not verified or not  reported | 2 | Medium-  Large | Poor to  Moderate |
|  | van Oosterhout et al. (2016) | Effects of metacognitive training on data  gathering bias | Effects of metacognitive training on data gathering bias | Randomised controlled trials | 0,307 |  |  |  | Small- Medium | [-0.16, 0.77] | No | N/A | 56,4 | No | 3 | 219 | No follow-up reported | Yes | Not verified or not reported | 1 | Small- Medium | Poor |

Note. **Conclusions on effect size.** d/g: < 0.2 = No effect or very small; 0.2 to < 0.3 = Small; 0. to < 0.45 = Small-Medium; 0.45 to < 0.55 = Medium; 0.55 to < 0.75 = Medium-Large; 0.75 to < 1 = Large; > 1 = Very Large. R or RS: < 0.1 = No effect or very small; 0.1 to < 0.2 = Small; 0.2 to < 0.3 = Medium; 0.3 to < 0.4 = Medium-Large; 0.4 to < 0.5 = Large; > 0.5 = Very Large. OR: < 1= No effect or very small; 1 to < 1.25 = Small; 1.25 to < 1.50 = Medium; 1.50 to < 2.50 = Medium-Large; 2.50 to < 10 = Large **Size of the sample.** < 500 = 0 point; 500 to < 1000 = 0.5 point, > 1000 = 1 point. **Precision of effects.** Large CIs > 0.25 in either direction = 0 point; Tight CIs < 0.25 in either direction = 1 point. **Homogeneity of effects across studies.** I2> 30% or Q is significant = 0 point; I2 < 30% or Q is not significant = 1 point. **Follow-up data.** Absence of follow-up data = 0 point; Presence of follow-up data (less than six months) = 0.5 point; Presence of follow-up data (six months or more) = 1 point. **Publication bias.** Not verified or not reported or verified and presence of publication bias = 0 point; Verified and absence of bias = 1 point. **Confounding factors.** No verified = 0 point; Verified = 1 point. **Overall Quality.** Total points for all elements of the GRADE system measured: < 1 = poor; 1 to < 2 =Poor to Moderate; 2 to < 3 = Moderate; 3 to < 4 = Moderate-High; 4 to 6 = High.
